# Supplementary material for: Transformative insights from transcriptome analysis of colorectal cancer patient tissues: identification of four key prognostic genes
Source: PeerJ. 2025 Aug 20;13:e19852. doi: 10.7717/peerj.19852 (PMC12374693; doi:10.7717/peerj.19852)
Supplement: Supplemental Information 1 [file peerj-13-19852-s001.docx]

**Supplementary Tables**

**Supplementary Table 1**. Summary of GEO mRNA microarray datasets used for integrated tumor–normal differential expression analysis

| **Dataset ID** | **Platform** | **Number of samples and clinical characteristics** | | **Sample Type** | **Year** | **Reference** |
| --- | --- | --- | --- | --- | --- | --- |
|  |  | **tumor** | **normal** |  |  |  |
| GSE9348 | Affymetrix Human Genome U133 Plus 2.0 Array | 70 | 12 | Unpaired | 2010 | PMID:20143136 |
| GSE21510 | Affymetrix Human Genome U133 Plus 2.0 Array | 123 | 25 | Unpaired | 2011 | PMID:21270110 |
|  |  | StageI:15, StageII:46, StageIII:39, StageIV:23 |  |  |  |  |
| GSE24550 | Affymetrix Human Exon 1.0 ST Array | 77 | 13 | Unpaired | 2011 | PMID: 21619627 |
|  |  | StageII:44, StageIII:43 MSI status:  MSI-L:10, MSI-H:14, MSS:41, NA:12 |  | Paired | 2015 | PMID: 26528635 |

* *This table summarizes the GEO mRNA datasets used for tumor-normal differential expression analysis, including platform, sample counts, and available clinical metadata.*

**Supplementary Table 2**. Summary of public miRNA microarray datasets used for integrated tumor–normal differential expression analysis

| **Dataset ID** | **Platform** | **Number of samples and clinical characteristics** | | **Sample Type** | **Year** | **Reference** |
| --- | --- | --- | --- | --- | --- | --- |
|  |  | **tumor** | **normal** |  |  |  |
| E-MTAB-752 | Affymetrix GeneChip miRNA 2.0 Array | 19 | 19 | Paired | 2012 | PMID:22479426 |
| E-GEOD-35834 | Affymetrix miRNA Array 2.0 | 31 primitive tumor + 24 liver metastases | 23 | Mixed (Paired & Unpaired) | 2013 | PMID:23987127 |
|  |  | Mean Age:61, Sex: Female:12, Male:26 Grade1:2 Grade2:23, Grade3:11, NA:2 StageI:2, StageII:1, StageIII:1, StageIV:33, NA:1  Location: Left-sided:28, Right-sided:10 | Mean Age:60, Sex: Female:11, Male:12 Location: Left-sided:17, Right-sided:6 |  |  |  |
| E-MTAB-813 | Agilent Human miRNA Microarray Kit (V3) 8x15K G4470C | 21 | 21 | Paired | 2011 | PMID: 21948089 |
| GSE68377 | Affymetrix GeneChip miRNA 1.0 array | 7 | 7 | Paired | 2015 | PMID: 26528635 |
| GSE35982 | Agilent- 021827 Human miRNA Microarray | 8 | 8 | Paired | 2013 | PMID: 22703586 |
|  |  | Mean Age:72, Sex: Female:5, Male:3 StageI:1, StageII:4, StageIII:3 Location: Left-sided:7, Right-sided:1 | |  |  |  |

**This table summarizes the public miRNA microarray datasets obtained from GEO and ArrayExpress, used for integrated tumor–normal differential expression analysis, including platform, sample counts, and available clinical metadata*

**Supplementary Table 3**. List of primers used for qRT-PCR

| **Gene Symbol** | **Forward Primer** | **Reverse Primer** | **Amplicon size (bp)** | **Accession No.** |
| --- | --- | --- | --- | --- |
| FN1 | GCTCTATTCCACCTTACAACAC | ACACAACGATGCTTCCTGAG | 155 | NM_212482.4 |
| COL1A1 | AAGAGGAAGGCCAAGTCGAG | AGATCACGTCATCGCACAAC | 156 | NM_000088.4 |
| COL5A1 | GATGTCGCTTACAGAGTCACC | TTGGCTTTCACAGTTGTTAGGA | 112 | NM_000093.5 |
| THBS2 | AGCTCAGCGAGAACCTCAAG | TTCATTTTCCGCAAAGAACC | 128 | NM_003247.5 |
| B2M | TGCTGTCTCCATGTTTGATGTATCT | TCTCTGCTCCCCACCTCTAAGT | 86 | NM_004048.4 |
| HPRT | TGACACTGGCAAAACAATGCA | GGTCCTTTTCACCAGCAAGCT | 94 | NM_000194.3 |

**Supplementary Table 4**. Complete list of differentially expressed genes obtained from transcriptome analysis of 49 colorectal cancer tissues and their matched normal tissue samples (discovery cohort)

| **Gene Symbol** | **p-value** | **adj.p-value** | **log2FC** |
| --- | --- | --- | --- |
| THBS2 | 8.23E-17 | 1.40E-13 | 4.34 |
| COL10A1 | 1.85E-14 | 1.00E-11 | 4.23 |
| FAP | 1.93E-18 | 8.44E-15 | 4.13 |
| INHBA | 1.35E-18 | 8.08E-15 | 4.06 |
| GRIN2D | 1.11E-16 | 1.70E-13 | 3.91 |
| MMP11 | 1.71E-13 | 5.84E-11 | 3.64 |
| SULF1 | 5.73E-17 | 1.13E-13 | 3.44 |
| BGN | 4.11E-18 | 1.58E-14 | 3.43 |
| SPP1 | 2.57E-11 | 3.49E-09 | 3.29 |
| H19 /// MIR675 /// RPS12 | 4.43E-14 | 2.00E-11 | 3.27 |
| COL1A2 | 2.54E-15 | 1.97E-12 | 3.14 |
| CXCL8 | 1.11E-09 | 7.87E-08 | 3.01 |
| MFAP2 | 2.49E-16 | 3.06E-13 | 2.97 |
| COL12A1 | 5.25E-14 | 2.28E-11 | 2.91 |
| COL1A1 | 9.01E-15 | 5.36E-12 | 2.90 |
| TIMP1 | 6.58E-15 | 4.12E-12 | 2.82 |
| CLDN1 | 6.37E-15 | 4.11E-12 | 2.69 |
| CST4 | 2.85E-13 | 9.07E-11 | 2.62 |
| CHI3L1 | 7.81E-09 | 4.13E-07 | 2.60 |
| COL11A1 | 1.50E-10 | 1.49E-08 | 2.58 |
| FNDC1 | 1.13E-12 | 2.65E-10 | 2.58 |
| CST1 | 4.54E-12 | 8.20E-10 | 2.55 |
| SFRP4 | 8.61E-10 | 6.29E-08 | 2.55 |
| CDKN2A | 2.28E-09 | 1.46E-07 | 2.53 |
| VCAN | 3.46E-15 | 2.56E-12 | 2.46 |
| DIO2 | 1.07E-12 | 2.55E-10 | 2.42 |
| VSNL1 | 2.72E-10 | 2.46E-08 | 2.39 |
| COL8A1 | 4.89E-11 | 5.94E-09 | 2.36 |
| FCGR3A /// FCGR3B | 5.57E-12 | 9.80E-10 | 2.35 |
| LEF1 | 7.99E-14 | 3.23E-11 | 2.31 |
| LTBP2 | 1.15E-13 | 4.30E-11 | 2.27 |
| DUXAP10 /// LINC01296 | 2.60E-11 | 3.53E-09 | 2.26 |
| BC017398 /// CTD-2314B22.3 /// DQ786293 /// DUXAP10 /// LL22NC03-N64E9.1 | 2.48E-14 | 1.27E-11 | 2.25 |
| THY1 | 6.81E-16 | 6.85E-13 | 2.23 |
| CDH11 | 1.65E-11 | 2.42E-09 | 2.19 |
| AZGP1 | 1.84E-09 | 1.22E-07 | 2.16 |
| ISLR | 1.83E-09 | 1.22E-07 | 2.12 |
| MMP1 | 1.66E-06 | 3.53E-05 | 2.11 |
| RAB31 | 1.34E-11 | 2.01E-09 | 2.07 |
| --- | 1.80E-14 | 9.85E-12 | 2.05 |
| ANTXR1 | 8.69E-11 | 9.47E-09 | 2.05 |
| ITGBL1 | 8.04E-08 | 2.86E-06 | 2.04 |
| PRRX1 | 6.49E-12 | 1.10E-09 | 2.03 |
| CTHRC1 | 6.92E-10 | 5.29E-08 | 1.95 |
| SPOCK1 | 5.89E-09 | 3.26E-07 | 1.94 |
| SFRP2 | 2.90E-07 | 8.27E-06 | 1.94 |
| ASPN | 3.99E-09 | 2.34E-07 | 1.93 |
| COL5A2 | 4.98E-11 | 6.01E-09 | 1.93 |
| GABBR1 /// UBD | 6.68E-10 | 5.14E-08 | 1.92 |
| SLC6A6 | 3.11E-13 | 9.68E-11 | 1.91 |
| COL5A1 | 2.78E-12 | 5.48E-10 | 1.90 |
| GUCY1A3 | 3.43E-10 | 2.98E-08 | 1.90 |
| TGFBI | 1.52E-12 | 3.39E-10 | 1.89 |
| MEGF6 | 8.94E-11 | 9.73E-09 | 1.88 |
| EDNRA | 6.28E-13 | 1.70E-10 | 1.88 |
| SERPINE1 | 3.45E-09 | 2.09E-07 | 1.86 |
| LGR5 | 5.70E-08 | 2.15E-06 | 1.85 |
| KLK10 | 2.10E-07 | 6.32E-06 | 1.85 |
| TDGF1 /// TDGF1P3 | 4.88E-07 | 1.27E-05 | 1.82 |
| RIPK2 | 8.25E-16 | 8.04E-13 | 1.81 |
| LOC100130872 /// SPON2 | 1.41E-13 | 5.02E-11 | 1.81 |
| HILPDA | 3.09E-13 | 9.67E-11 | 1.80 |
| SOX4 | 1.10E-16 | 1.70E-13 | 1.79 |
| FOXQ1 | 9.02E-09 | 4.68E-07 | 1.78 |
| COL3A1 | 2.55E-11 | 3.48E-09 | 1.78 |
| MMP14 | 2.91E-12 | 5.67E-10 | 1.76 |
| SPARC | 6.85E-08 | 2.50E-06 | 1.76 |
| OLFML2B | 8.48E-13 | 2.16E-10 | 1.75 |
| CXCL9 | 1.84E-06 | 3.86E-05 | 1.75 |
| H19 /// MIR675 | 1.65E-08 | 7.71E-07 | 1.73 |
| LOXL2 | 3.10E-10 | 2.73E-08 | 1.71 |
| RGS16 | 1.85E-11 | 2.68E-09 | 1.71 |
| PLAU | 7.03E-10 | 5.35E-08 | 1.70 |
| HTRA1 | 1.94E-11 | 2.75E-09 | 1.70 |
| GPX8 | 1.46E-09 | 1.00E-07 | 1.70 |
| COL4A1 | 6.31E-11 | 7.29E-09 | 1.69 |
| LINC00152 /// LOC101930489 /// MIR4435-1HG | 3.82E-13 | 1.13E-10 | 1.67 |
| TGM2 | 5.31E-09 | 3.00E-07 | 1.66 |
| BCAT1 | 9.06E-09 | 4.70E-07 | 1.63 |
| THBS4 | 1.30E-06 | 2.91E-05 | 1.63 |
| TRIM29 | 3.07E-07 | 8.62E-06 | 1.62 |
| ADAMTS2 | 2.27E-11 | 3.15E-09 | 1.61 |
| SCD | 2.69E-07 | 7.80E-06 | 1.61 |
| HOTS | 6.32E-10 | 4.90E-08 | 1.60 |
| GTF2IRD1 | 1.28E-09 | 9.00E-08 | 1.60 |
| UCA1 | 5.32E-05 | 0.00062065 | 1.60 |
| WISP1 | 5.88E-09 | 3.26E-07 | 1.59 |
| GNG4 | 7.52E-06 | 0.00012347 | 1.59 |
| ETV5 | 3.66E-09 | 2.18E-07 | 1.59 |
| HOPX | 7.08E-10 | 5.37E-08 | 1.59 |
| AEBP1 | 9.62E-10 | 6.94E-08 | 1.57 |
| APOC1 | 5.76E-08 | 2.16E-06 | 1.57 |
| VEGFA | 9.63E-07 | 2.27E-05 | 1.57 |
| ENC1 | 2.56E-13 | 8.32E-11 | 1.56 |
| FBN1 | 4.81E-11 | 5.87E-09 | 1.56 |
| LUM | 4.90E-08 | 1.90E-06 | 1.56 |
| CTSK | 1.72E-09 | 1.15E-07 | 1.56 |
| IGFBP3 | 4.69E-10 | 3.84E-08 | 1.55 |
| C10orf10 | 3.96E-10 | 3.39E-08 | 1.55 |
| AHNAK2 | 1.09E-06 | 2.51E-05 | 1.55 |
| DPYSL3 | 5.57E-09 | 3.12E-07 | 1.53 |
| TWIST1 | 3.51E-09 | 2.11E-07 | 1.53 |
| TESC | 7.40E-10 | 5.58E-08 | 1.52 |
| ZFAS1 | 5.26E-11 | 6.25E-09 | 1.52 |
| LOC100506403 /// LOC101928269 /// RUNX1 | 3.40E-15 | 2.54E-12 | 1.52 |
| MGP | 2.37E-06 | 4.76E-05 | 1.52 |
| SERPINE2 | 2.71E-09 | 1.69E-07 | 1.51 |
| COL4A2 | 6.94E-12 | 1.16E-09 | 1.50 |
| FOXC1 | 1.02E-08 | 5.24E-07 | 1.50 |
| NOTCH3 | 5.81E-13 | 1.63E-10 | 1.50 |
| FXYD5 | 3.14E-11 | 4.16E-09 | 1.49 |
| MX1 | 3.20E-08 | 1.33E-06 | 1.48 |
| IGFBP5 | 8.41E-08 | 2.97E-06 | 1.48 |
| CITED4 | 6.56E-11 | 7.49E-09 | 1.47 |
| PLS3 | 1.50E-09 | 1.02E-07 | 1.47 |
| ELN | 2.56E-07 | 7.47E-06 | 1.46 |
| SERPINH1 | 1.01E-11 | 1.58E-09 | 1.45 |
| ADAMTS12 | 1.21E-10 | 1.23E-08 | 1.45 |
| C3 | 9.49E-07 | 2.23E-05 | 1.45 |
| ZAK | 3.69E-07 | 1.00E-05 | 1.45 |
| ASCL2 | 3.57E-10 | 3.08E-08 | 1.44 |
| ZNF117 | 7.77E-08 | 2.78E-06 | 1.43 |
| FN1 | 3.53E-09 | 2.12E-07 | 1.43 |
| NREP | 1.49E-10 | 1.49E-08 | 1.43 |
| ATP11A | 2.87E-10 | 2.56E-08 | 1.43 |
| TPM2 | 5.12E-10 | 4.10E-08 | 1.42 |
| KIRREL | 4.09E-10 | 3.47E-08 | 1.42 |
| TIMP3 | 2.00E-07 | 6.08E-06 | 1.42 |
| PDLIM7 | 3.02E-06 | 5.86E-05 | 1.42 |
| CYTH2 | 4.00E-07 | 1.07E-05 | 1.41 |
| CERCAM | 3.66E-09 | 2.18E-07 | 1.41 |
| PCOLCE | 5.78E-07 | 1.47E-05 | 1.41 |
| MMP3 | 6.68E-05 | 0.00074794 | 1.40 |
| LOC340340 | 3.34E-06 | 6.35E-05 | 1.40 |
| MXRA5 | 8.96E-07 | 2.13E-05 | 1.39 |
| CSGALNACT2 | 2.55E-11 | 3.48E-09 | 1.38 |
| SOX9 | 5.18E-08 | 1.99E-06 | 1.38 |
| IGF2 /// INS-IGF2 | 3.57E-08 | 1.45E-06 | 1.38 |
| TRPM2-AS | 2.94E-11 | 3.91E-09 | 1.37 |
| SNTB1 | 5.81E-10 | 4.57E-08 | 1.36 |
| MDK | 3.50E-08 | 1.43E-06 | 1.36 |
| IFI6 | 1.59E-14 | 8.97E-12 | 1.36 |
| MACC1 | 3.38E-09 | 2.05E-07 | 1.36 |
| TACSTD2 | 5.58E-07 | 1.43E-05 | 1.36 |
| PDGFRB | 4.72E-10 | 3.87E-08 | 1.35 |
| CNN2 | 4.20E-16 | 4.44E-13 | 1.35 |
| IFITM3 | 2.16E-10 | 2.02E-08 | 1.34 |
| MAML3 | 1.09E-08 | 5.50E-07 | 1.34 |
| CBX3 | 1.06E-10 | 1.11E-08 | 1.34 |
| NRP2 | 5.50E-08 | 2.09E-06 | 1.33 |
| COL6A3 | 4.35E-08 | 1.73E-06 | 1.33 |
| PMEPA1 | 2.23E-08 | 9.82E-07 | 1.33 |
| PDPN | 3.72E-06 | 6.92E-05 | 1.33 |
| NEBL | 1.71E-06 | 3.62E-05 | 1.32 |
| TPX2 | 1.26E-05 | 0.0001897 | 1.32 |
| POSTN | 4.83E-06 | 8.57E-05 | 1.32 |
| LOC102725271 /// NTM | 2.27E-08 | 1.00E-06 | 1.31 |
| CDC25B | 1.04E-10 | 1.10E-08 | 1.31 |
| CKS2 | 9.58E-06 | 0.0001513 | 1.31 |
| MMP2 | 1.93E-07 | 5.92E-06 | 1.31 |
| UBE2C | 4.19E-06 | 7.63E-05 | 1.31 |
| PFDN4 | 6.25E-12 | 1.08E-09 | 1.30 |
| GDF15 | 4.93E-07 | 1.28E-05 | 1.30 |
| XPO5 | 8.43E-08 | 2.97E-06 | 1.30 |
| CEMIP | 3.10E-08 | 1.30E-06 | 1.30 |
| GAS1 | 2.58E-06 | 5.12E-05 | 1.29 |
| TSHZ2 | 2.67E-07 | 7.74E-06 | 1.28 |
| CALU | 1.14E-07 | 3.81E-06 | 1.28 |
| SNHG6 /// SNORD87 | 3.61E-11 | 4.66E-09 | 1.28 |
| CDH3 | 3.57E-11 | 4.64E-09 | 1.28 |
| ARL4C | 1.67E-08 | 7.79E-07 | 1.28 |
| SYTL4 | 1.94E-07 | 5.95E-06 | 1.28 |
| PLXDC2 | 2.71E-07 | 7.83E-06 | 1.28 |
| SALL4 | 7.22E-11 | 8.09E-09 | 1.27 |
| HSP90AB1 | 9.38E-13 | 2.36E-10 | 1.27 |
| ANKRD10 | 5.27E-09 | 2.98E-07 | 1.27 |
| FOXK1 | 6.53E-13 | 1.75E-10 | 1.26 |
| DSCC1 | 4.60E-05 | 0.00055052 | 1.26 |
| GTF3A | 3.63E-09 | 2.17E-07 | 1.25 |
| MSX2 | 3.55E-06 | 6.66E-05 | 1.24 |
| ACTN1 | 3.26E-10 | 2.84E-08 | 1.24 |
| AXIN2 | 2.44E-06 | 4.87E-05 | 1.24 |
| PHLDA1 | 6.64E-06 | 0.00011172 | 1.24 |
| IFI44L | 4.17E-07 | 1.11E-05 | 1.24 |
| MET | 5.03E-06 | 8.86E-05 | 1.23 |
| CCND1 | 2.36E-11 | 3.25E-09 | 1.23 |
| LYZ | 3.27E-05 | 0.00041723 | 1.21 |
| DNAH14 | 6.45E-09 | 3.52E-07 | 1.21 |
| STMN3 | 2.22E-08 | 9.81E-07 | 1.20 |
| APBB2 | 3.70E-11 | 4.74E-09 | 1.20 |
| FCGR2A /// FCGR2C | 2.71E-07 | 7.85E-06 | 1.20 |
| CASC15 | 5.84E-08 | 2.19E-06 | 1.20 |
| CSE1L | 1.87E-06 | 3.91E-05 | 1.19 |
| UBE2T | 3.30E-07 | 9.17E-06 | 1.19 |
| HSPH1 | 1.93E-10 | 1.86E-08 | 1.19 |
| ITGA11 | 4.38E-07 | 1.16E-05 | 1.19 |
| DIXDC1 | 1.89E-06 | 3.94E-05 | 1.19 |
| NKD2 | 1.13E-07 | 3.79E-06 | 1.19 |
| FJX1 | 3.86E-08 | 1.56E-06 | 1.18 |
| NQO1 | 8.10E-06 | 0.00013159 | 1.18 |
| XAF1 | 1.90E-07 | 5.85E-06 | 1.18 |
| OSBPL8 | 1.20E-08 | 5.93E-07 | 1.18 |
| BCL2L1 | 1.07E-10 | 1.12E-08 | 1.18 |
| IFIT3 | 8.32E-07 | 2.00E-05 | 1.18 |
| PLEKHS1 | 6.12E-07 | 1.55E-05 | 1.17 |
| STAT1 | 1.79E-08 | 8.26E-07 | 1.17 |
| REXO2 | 3.51E-08 | 1.44E-06 | 1.17 |
| STC2 | 7.66E-08 | 2.75E-06 | 1.17 |
| NR1D1 /// THRA | 1.02E-07 | 3.49E-06 | 1.17 |
| TTYH3 | 3.74E-10 | 3.22E-08 | 1.16 |
| PALD1 | 1.38E-11 | 2.06E-09 | 1.16 |
| WNT5A | 1.36E-05 | 0.00020128 | 1.16 |
| SMARCA4 | 7.05E-07 | 1.74E-05 | 1.16 |
| NAP1L1 | 3.34E-12 | 6.30E-10 | 1.16 |
| AC017002.2 | 2.31E-08 | 1.01E-06 | 1.15 |
| ATAD2 | 1.43E-11 | 2.12E-09 | 1.15 |
| MXRA8 | 1.67E-07 | 5.25E-06 | 1.15 |
| EVC | 1.75E-07 | 5.48E-06 | 1.15 |
| DUSP4 | 2.09E-07 | 6.32E-06 | 1.14 |
| IFITM2 | 1.49E-09 | 1.02E-07 | 1.14 |
| PLA2G16 | 4.89E-08 | 1.90E-06 | 1.14 |
| TBC1D16 | 2.49E-08 | 1.08E-06 | 1.14 |
| TIMP2 | 7.26E-07 | 1.78E-05 | 1.14 |
| PPFIBP1 | 9.49E-06 | 0.0001501 | 1.14 |
| CYP2S1 | 1.15E-06 | 2.61E-05 | 1.14 |
| LOC101928916 /// NNMT | 3.78E-07 | 1.03E-05 | 1.14 |
| HSPD1 | 3.33E-09 | 2.03E-07 | 1.14 |
| WWC3 | 6.73E-10 | 5.16E-08 | 1.14 |
| RCN3 | 1.05E-05 | 0.00016346 | 1.14 |
| AP5Z1 /// FOXK1 /// MIR4656 | 2.87E-08 | 1.22E-06 | 1.13 |
| DKC1 /// MIR664B /// SNORA56 | 2.52E-07 | 7.39E-06 | 1.13 |
| ITGB5 | 2.08E-07 | 6.29E-06 | 1.13 |
| PROX1 | 1.41E-06 | 3.10E-05 | 1.13 |
| LGALS1 | 6.02E-10 | 4.70E-08 | 1.13 |
| C16orf13 | 1.41E-12 | 3.18E-10 | 1.13 |
| GXYLT2 | 7.66E-05 | 0.00083365 | 1.13 |
| MRC2 | 2.91E-07 | 8.29E-06 | 1.13 |
| PLXDC1 | 2.52E-08 | 1.09E-06 | 1.13 |
| ASPM | 6.55E-09 | 3.56E-07 | 1.12 |
| TNS4 | 1.84E-08 | 8.46E-07 | 1.12 |
| TSPAN9 | 9.87E-08 | 3.40E-06 | 1.12 |
| MSR1 | 3.86E-08 | 1.56E-06 | 1.12 |
| COL27A1 | 3.39E-07 | 9.39E-06 | 1.11 |
| HSPG2 | 2.20E-08 | 9.73E-07 | 1.11 |
| OLR1 | 7.59E-07 | 1.86E-05 | 1.11 |
| DKK3 | 7.10E-07 | 1.75E-05 | 1.11 |
| CYP1B1 | 1.74E-06 | 3.69E-05 | 1.11 |
| MSX1 | 1.54E-07 | 4.93E-06 | 1.11 |
| VPS9D1-AS1 | 2.50E-07 | 7.35E-06 | 1.11 |
| RP11-803D5.4 | 3.51E-09 | 2.11E-07 | 1.11 |
| CTSH | 5.69E-08 | 2.14E-06 | 1.10 |
| PTPN14 | 4.13E-10 | 3.48E-08 | 1.10 |
| CALD1 | 1.83E-05 | 0.00025704 | 1.10 |
| TRAF5 | 1.31E-09 | 9.17E-08 | 1.10 |
| EPSTI1 | 1.90E-08 | 8.64E-07 | 1.10 |
| AP000525.9 | 1.23E-05 | 0.00018551 | 1.10 |
| RAI14 | 1.70E-05 | 0.00024249 | 1.09 |
| SLCO4A1 | 2.44E-07 | 7.20E-06 | 1.09 |
| NNMT | 4.86E-07 | 1.27E-05 | 1.09 |
| FAM60A | 4.53E-09 | 2.60E-07 | 1.09 |
| PTGIS | 3.45E-06 | 6.54E-05 | 1.09 |
| ATHL1 | 4.30E-07 | 1.14E-05 | 1.09 |
| YAP1 | 3.24E-12 | 6.18E-10 | 1.08 |
| RCL1 | 1.61E-12 | 3.58E-10 | 1.08 |
| CDKN3 | 3.03E-05 | 0.00039103 | 1.08 |
| TGFB2 | 6.94E-08 | 2.53E-06 | 1.08 |
| KLF7 | 5.30E-08 | 2.03E-06 | 1.08 |
| GBP4 | 7.46E-06 | 0.00012265 | 1.08 |
| EIF2S3 | 2.43E-08 | 1.06E-06 | 1.08 |
| FADS1 /// MIR1908 | 3.25E-06 | 6.21E-05 | 1.07 |
| S100A11 | 8.16E-12 | 1.32E-09 | 1.07 |
| FSTL1 | 2.74E-06 | 5.38E-05 | 1.06 |
| LIMS1 /// LIMS3L | 4.28E-10 | 3.58E-08 | 1.06 |
| C1QTNF5 /// MFRP | 2.14E-09 | 1.39E-07 | 1.06 |
| LOC102725263 /// LOC389906 | 9.38E-05 | 0.00098413 | 1.06 |
| RBCK1 | 3.19E-07 | 8.92E-06 | 1.06 |
| BMP4 | 2.78E-06 | 5.45E-05 | 1.06 |
| ANKRD13B | 3.39E-07 | 9.39E-06 | 1.05 |
| STC1 | 5.97E-07 | 1.51E-05 | 1.05 |
| MOXD1 | 4.16E-06 | 7.58E-05 | 1.05 |
| NELFCD | 1.75E-08 | 8.08E-07 | 1.05 |
| CPNE1 | 8.50E-10 | 6.25E-08 | 1.05 |
| COMP | 7.03E-06 | 0.00011701 | 1.05 |
| FMNL2 | 2.07E-10 | 1.96E-08 | 1.05 |
| IFITM1 | 2.22E-09 | 1.43E-07 | 1.04 |
| LOC100996643 /// LOC101928195 /// MTHFD1L | 6.13E-10 | 4.77E-08 | 1.04 |
| CASP4 | 9.97E-06 | 0.00015647 | 1.04 |
| KRT80 | 1.58E-08 | 7.41E-07 | 1.04 |
| GFPT2 | 3.98E-06 | 7.33E-05 | 1.04 |
| NPM1 | 1.65E-06 | 3.51E-05 | 1.04 |
| MIR1304 /// SNORA1 /// SNORA18 /// SNORA32 /// SNORA40 /// SNORA8 /// SNORD5 /// TAF1D | 2.21E-07 | 6.58E-06 | 1.03 |
| ELK1 | 2.12E-09 | 1.37E-07 | 1.03 |
| SULF2 | 5.38E-05 | 0.00062605 | 1.03 |
| CD44 | 2.12E-07 | 6.38E-06 | 1.03 |
| PLXNA1 | 4.42E-10 | 3.67E-08 | 1.03 |
| PHF20 | 5.90E-09 | 3.26E-07 | 1.03 |
| LOC100129518 /// SOD2 | 1.12E-06 | 2.57E-05 | 1.03 |
| FKBP10 | 1.07E-08 | 5.43E-07 | 1.03 |
| SDCCAG3 | 3.13E-06 | 6.03E-05 | 1.03 |
| LAMB2 | 3.36E-06 | 6.40E-05 | 1.03 |
| TRNP1 | 1.38E-05 | 0.00020389 | 1.03 |
| IFITM1 /// IFITM2 | 1.87E-08 | 8.56E-07 | 1.02 |
| IQGAP3 | 4.86E-06 | 8.61E-05 | 1.02 |
| ZNF503 | 6.08E-06 | 0.00010412 | 1.02 |
| HSP90AA1 | 6.00E-09 | 3.31E-07 | 1.02 |
| ANGPTL2 | 6.53E-07 | 1.63E-05 | 1.02 |
| PRKDC | 7.48E-07 | 1.83E-05 | 1.01 |
| CAMSAP2 | 1.53E-11 | 2.26E-09 | 1.01 |
| TOP2A | 2.62E-05 | 0.00034691 | 1.01 |
| SORD | 1.09E-05 | 0.00016801 | 1.01 |
| TKT | 6.65E-06 | 0.00011189 | 1.01 |
| PLCB1 | 2.34E-05 | 0.00031564 | 1.01 |
| PCNXL2 | 3.94E-08 | 1.59E-06 | 1.01 |
| F2R | 3.94E-08 | 1.59E-06 | 1.01 |
| MCFD2 | 3.51E-07 | 9.66E-06 | 1.01 |
| KCNAB2 | 2.90E-07 | 8.26E-06 | 1.00 |
| GTF2I /// GTF2IP1 /// LOC100093631 | 6.21E-15 | 4.05E-12 | 1.00 |
| DCAF13 | 9.36E-08 | 3.26E-06 | 1.00 |
| DBN1 | 4.84E-10 | 3.94E-08 | 1.00 |
| LZTS1 | 3.23E-09 | 1.98E-07 | 1.00 |
| CCNYL1 | 7.47E-12 | 1.22E-09 | -1.01 |
| EMID1 | 6.52E-08 | 2.40E-06 | -1.01 |
| TCF21 | 3.25E-10 | 2.83E-08 | -1.01 |
| MAGI3 | 1.56E-07 | 4.99E-06 | -1.01 |
| AP5M1 | 8.74E-10 | 6.36E-08 | -1.01 |
| PTP4A1 | 8.49E-12 | 1.36E-09 | -1.01 |
| CEACAM5 | 3.15E-05 | 0.0004037 | -1.01 |
| LINC01207 | 2.50E-07 | 7.34E-06 | -1.01 |
| BTG2 | 2.33E-09 | 1.48E-07 | -1.01 |
| FAM107A | 1.27E-10 | 1.29E-08 | -1.01 |
| IGLV@ | 1.37E-06 | 3.04E-05 | -1.01 |
| SNORD3A /// SNORD3B-1 /// SNORD3B-2 /// SNORD3C /// SNORD3D | 4.49E-09 | 2.59E-07 | -1.01 |
| CAMK2D | 5.56E-09 | 3.12E-07 | -1.02 |
| NHEJ1 /// SLC23A3 | 7.57E-07 | 1.85E-05 | -1.02 |
| FRYL | 2.57E-08 | 1.11E-06 | -1.02 |
| AMELX | 6.66E-05 | 0.00074661 | -1.02 |
| ELP5 | 6.98E-09 | 3.76E-07 | -1.02 |
| P2RX1 | 4.18E-10 | 3.51E-08 | -1.02 |
| PPARD | 2.28E-10 | 2.12E-08 | -1.02 |
| PRELID2 | 8.23E-09 | 4.33E-07 | -1.02 |
| AVPI1 | 9.00E-07 | 2.13E-05 | -1.02 |
| CDH19 | 7.68E-08 | 2.76E-06 | -1.02 |
| FGL2 | 3.49E-09 | 2.11E-07 | -1.03 |
| FAM134B | 4.68E-08 | 1.83E-06 | -1.03 |
| TCF7L2 | 7.03E-10 | 5.35E-08 | -1.03 |
| TMEM37 | 1.06E-07 | 3.60E-06 | -1.03 |
| RALGAPA1 | 1.27E-08 | 6.21E-07 | -1.03 |
| LOC101927157 /// NIPAL1 | 1.34E-08 | 6.46E-07 | -1.03 |
| CCL13 | 8.75E-06 | 0.0001402 | -1.03 |
| FEM1C | 5.21E-07 | 1.35E-05 | -1.03 |
| SLC45A3 | 8.13E-07 | 1.96E-05 | -1.03 |
| C14orf182 | 2.48E-08 | 1.08E-06 | -1.03 |
| NEDD4L | 1.20E-12 | 2.76E-10 | -1.03 |
| TMEM171 | 1.06E-12 | 2.54E-10 | -1.03 |
| ACSBG1 | 1.02E-05 | 0.00015903 | -1.03 |
| MOB3B | 5.07E-11 | 6.08E-09 | -1.03 |
| PHYKPL | 8.06E-15 | 4.95E-12 | -1.04 |
| MYOT | 3.73E-09 | 2.22E-07 | -1.04 |
| ZNF655 | 7.51E-12 | 1.23E-09 | -1.04 |
| FCRL5 | 5.04E-07 | 1.31E-05 | -1.04 |
| CD209 | 4.30E-06 | 7.80E-05 | -1.04 |
| FMO5 | 5.95E-10 | 4.66E-08 | -1.04 |
| ZSWIM6 | 6.49E-12 | 1.10E-09 | -1.04 |
| PPARGC1B | 6.06E-12 | 1.06E-09 | -1.04 |
| ST6GALNAC6 | 1.30E-11 | 1.96E-09 | -1.04 |
| CXCL14 | 6.87E-05 | 0.00076273 | -1.05 |
| PRO0471 | 2.94E-10 | 2.61E-08 | -1.05 |
| SPDEF | 1.89E-07 | 5.82E-06 | -1.05 |
| STXBP5-AS1 | 1.74E-08 | 8.07E-07 | -1.05 |
| SCN7A | 5.38E-10 | 4.28E-08 | -1.05 |
| HK2 /// RP11-259N19.1 | 2.48E-08 | 1.08E-06 | -1.05 |
| TSPAN3 | 5.49E-09 | 3.08E-07 | -1.05 |
| MT1F | 1.88E-05 | 0.00026337 | -1.05 |
| FKBP1B | 1.37E-06 | 3.04E-05 | -1.05 |
| MXI1 | 9.81E-08 | 3.39E-06 | -1.05 |
| NFATC3 | 8.52E-07 | 2.04E-05 | -1.05 |
| RBM47 | 1.85E-08 | 8.48E-07 | -1.05 |
| PGM1 | 4.21E-08 | 1.68E-06 | -1.05 |
| TRIM40 | 2.87E-05 | 0.00037397 | -1.06 |
| MUC3B | 2.38E-05 | 0.00032041 | -1.06 |
| CTSS | 1.30E-12 | 2.95E-10 | -1.06 |
| LGR4 | 8.54E-10 | 6.28E-08 | -1.06 |
| IGKC | 2.16E-05 | 0.00029453 | -1.06 |
| PACSIN2 | 4.99E-11 | 6.01E-09 | -1.06 |
| FAM84A | 1.66E-05 | 0.00023861 | -1.06 |
| NHSL1 | 1.01E-07 | 3.47E-06 | -1.06 |
| UBE2J1 | 1.75E-10 | 1.71E-08 | -1.06 |
| SIPA1L2 | 1.44E-13 | 5.06E-11 | -1.06 |
| TFCP2L1 | 1.05E-12 | 2.54E-10 | -1.06 |
| WNT2B | 2.24E-06 | 4.57E-05 | -1.06 |
| JAM2 | 6.07E-07 | 1.54E-05 | -1.06 |
| FAM46A | 5.53E-08 | 2.10E-06 | -1.06 |
| KLRB1 | 6.20E-09 | 3.40E-07 | -1.06 |
| CP | 1.20E-10 | 1.23E-08 | -1.07 |
| PRAC1 | 9.04E-07 | 2.14E-05 | -1.07 |
| DMXL1 | 1.06E-09 | 7.55E-08 | -1.07 |
| SUCLG2 | 5.91E-08 | 2.21E-06 | -1.07 |
| FOS | 5.74E-08 | 2.16E-06 | -1.07 |
| FBXL14 | 9.32E-11 | 1.00E-08 | -1.07 |
| EFCAB4B | 2.50E-08 | 1.08E-06 | -1.07 |
| LOC100506558 /// MATN2 | 1.45E-11 | 2.14E-09 | -1.07 |
| TPMT | 2.65E-07 | 7.69E-06 | -1.07 |
| SYTL2 | 1.51E-06 | 3.27E-05 | -1.07 |
| CPM | 1.38E-13 | 4.95E-11 | -1.07 |
| RHOF | 8.35E-07 | 2.01E-05 | -1.07 |
| HSD17B11 | 3.56E-09 | 2.13E-07 | -1.07 |
| CASP1 | 2.69E-10 | 2.43E-08 | -1.08 |
| LINC00969 | 3.98E-10 | 3.40E-08 | -1.08 |
| PDE8A | 7.82E-10 | 5.84E-08 | -1.08 |
| SSBP2 | 2.53E-07 | 7.41E-06 | -1.08 |
| SFRP1 | 1.35E-08 | 6.53E-07 | -1.08 |
| ZZEF1 | 1.98E-12 | 4.19E-10 | -1.08 |
| PLCE1 | 7.27E-13 | 1.90E-10 | -1.08 |
| FBXO34 | 1.18E-08 | 5.85E-07 | -1.08 |
| AK057259 /// AK096803 /// RP11-395B7.2 | 4.72E-07 | 1.24E-05 | -1.08 |
| NEO1 | 4.28E-13 | 1.24E-10 | -1.08 |
| IGK | 4.74E-10 | 3.88E-08 | -1.08 |
| TRIB1 | 1.32E-08 | 6.42E-07 | -1.09 |
| CACNB2 | 3.55E-09 | 2.13E-07 | -1.09 |
| TMEM54 | 2.19E-09 | 1.41E-07 | -1.09 |
| HNF4G | 5.52E-08 | 2.09E-06 | -1.09 |
| HMGCR | 1.32E-06 | 2.94E-05 | -1.09 |
| SCP2 | 6.62E-08 | 2.43E-06 | -1.09 |
| PRR5L | 3.90E-09 | 2.29E-07 | -1.09 |
| BEST4 | 1.54E-07 | 4.93E-06 | -1.09 |
| CALML4 | 4.41E-08 | 1.75E-06 | -1.09 |
| PRKACB | 3.21E-11 | 4.23E-09 | -1.09 |
| CPA3 | 1.26E-08 | 6.18E-07 | -1.09 |
| TPD52 | 3.27E-09 | 2.00E-07 | -1.10 |
| PLXNA2 | 4.19E-08 | 1.68E-06 | -1.10 |
| HRASLS2 | 4.41E-07 | 1.17E-05 | -1.10 |
| RHBDL2 | 1.03E-08 | 5.27E-07 | -1.10 |
| TADA2B | 1.13E-08 | 5.66E-07 | -1.10 |
| AFG3L2 | 2.11E-07 | 6.36E-06 | -1.10 |
| RASEF | 2.48E-08 | 1.08E-06 | -1.11 |
| C11orf54 | 1.51E-08 | 7.15E-07 | -1.11 |
| ATP2B1 | 2.52E-12 | 5.10E-10 | -1.11 |
| CDC26 | 1.87E-09 | 1.24E-07 | -1.11 |
| TSPAN8 | 1.00E-07 | 3.44E-06 | -1.11 |
| UQCRFS1 | 1.19E-07 | 3.96E-06 | -1.11 |
| CCDC69 | 4.30E-08 | 1.72E-06 | -1.11 |
| FGD4 | 1.76E-10 | 1.72E-08 | -1.11 |
| MIER3 | 1.94E-09 | 1.28E-07 | -1.11 |
| ATP8A1 | 4.52E-11 | 5.58E-09 | -1.11 |
| PPP1R14D | 4.39E-08 | 1.74E-06 | -1.12 |
| RP11-747H7.3 | 5.82E-08 | 2.18E-06 | -1.12 |
| KIF13B | 5.81E-11 | 6.84E-09 | -1.12 |
| B3GALT1 | 7.84E-10 | 5.84E-08 | -1.12 |
| TCEA3 | 1.82E-07 | 5.66E-06 | -1.12 |
| DSG2 | 1.21E-05 | 0.00018365 | -1.13 |
| C1orf115 | 7.00E-10 | 5.33E-08 | -1.13 |
| KLF3 | 9.01E-10 | 6.55E-08 | -1.13 |
| TMEM45B | 4.02E-08 | 1.62E-06 | -1.13 |
| TMCC3 | 5.89E-11 | 6.91E-09 | -1.13 |
| GSKIP | 3.61E-06 | 6.76E-05 | -1.13 |
| LGALS3 | 1.07E-08 | 5.42E-07 | -1.13 |
| LRMP | 1.55E-06 | 3.35E-05 | -1.13 |
| LOC283070 | 1.57E-07 | 4.99E-06 | -1.13 |
| MTMR11 | 7.14E-08 | 2.60E-06 | -1.13 |
| C5orf30 | 6.76E-11 | 7.71E-09 | -1.14 |
| GALNT5 | 1.04E-06 | 2.42E-05 | -1.14 |
| ACVRL1 | 1.17E-08 | 5.82E-07 | -1.14 |
| GOLM1 | 4.98E-10 | 4.02E-08 | -1.14 |
| PDE3A | 5.49E-08 | 2.09E-06 | -1.14 |
| ENTPD5 | 2.30E-10 | 2.13E-08 | -1.14 |
| ATP2A3 | 1.62E-08 | 7.59E-07 | -1.14 |
| CLMN | 1.34E-11 | 2.01E-09 | -1.14 |
| KIAA1324 | 5.18E-05 | 0.00060615 | -1.14 |
| CPNE8 | 4.08E-10 | 3.47E-08 | -1.14 |
| MYH11 | 4.81E-05 | 0.00057124 | -1.14 |
| GPM6B | 1.02E-11 | 1.59E-09 | -1.15 |
| RIOK3 | 3.64E-09 | 2.18E-07 | -1.15 |
| GSR | 6.35E-07 | 1.60E-05 | -1.15 |
| FGFR2 | 1.07E-06 | 2.47E-05 | -1.15 |
| C1orf210 | 7.92E-10 | 5.88E-08 | -1.15 |
| GGT6 | 3.39E-14 | 1.56E-11 | -1.15 |
| CNST | 1.59E-10 | 1.57E-08 | -1.15 |
| ANGPTL1 | 5.19E-12 | 9.17E-10 | -1.15 |
| MYO5C | 1.99E-07 | 6.06E-06 | -1.15 |
| SLC39A8 | 8.41E-09 | 4.40E-07 | -1.15 |
| EXPH5 | 7.77E-07 | 1.89E-05 | -1.16 |
| CLINT1 | 9.56E-11 | 1.02E-08 | -1.16 |
| TRPM6 | 9.71E-08 | 3.37E-06 | -1.16 |
| CHP1 | 7.04E-11 | 7.94E-09 | -1.16 |
| RP11-845C23.3 | 6.16E-11 | 7.17E-09 | -1.16 |
| CYAT1 /// IGLC1 /// IGLV1-44 | 4.27E-06 | 7.75E-05 | -1.16 |
| ABCC3 | 4.36E-07 | 1.16E-05 | -1.16 |
| PTGDR | 4.39E-10 | 3.65E-08 | -1.16 |
| MGLL | 3.58E-08 | 1.46E-06 | -1.16 |
| NR5A2 | 3.94E-12 | 7.35E-10 | -1.16 |
| GLIPR2 | 1.40E-08 | 6.72E-07 | -1.16 |
| STARD10 | 8.12E-10 | 6.01E-08 | -1.16 |
| PDK4 | 4.12E-10 | 3.48E-08 | -1.17 |
| IGLL3P | 2.14E-08 | 9.56E-07 | -1.17 |
| MT1G | 6.48E-11 | 7.43E-09 | -1.17 |
| HHLA2 | 6.16E-10 | 4.79E-08 | -1.17 |
| RAB27A | 5.51E-10 | 4.37E-08 | -1.17 |
| EMP1 | 8.70E-09 | 4.54E-07 | -1.17 |
| KLF9 | 5.75E-11 | 6.79E-09 | -1.18 |
| BCL2L14 | 2.10E-06 | 4.33E-05 | -1.18 |
| MUC13 | 4.83E-10 | 3.93E-08 | -1.18 |
| SH3PXD2A | 1.16E-09 | 8.17E-08 | -1.18 |
| AKAP7 | 1.13E-07 | 3.79E-06 | -1.18 |
| MCTP2 | 2.12E-13 | 7.08E-11 | -1.18 |
| CGN | 2.15E-10 | 2.02E-08 | -1.18 |
| GAB1 | 1.09E-09 | 7.73E-08 | -1.18 |
| LRRC1 | 4.93E-07 | 1.28E-05 | -1.19 |
| BCL10 | 1.16E-08 | 5.78E-07 | -1.19 |
| CCDC152 | 7.33E-06 | 0.00012103 | -1.19 |
| LIMA1 | 3.04E-11 | 4.03E-09 | -1.19 |
| ABI3BP | 9.54E-11 | 1.02E-08 | -1.19 |
| TSC22D3 | 3.20E-10 | 2.80E-08 | -1.19 |
| EDIL3 | 1.61E-06 | 3.46E-05 | -1.19 |
| SCNN1A | 6.94E-06 | 0.00011564 | -1.19 |
| AKR1C3 | 2.85E-06 | 5.57E-05 | -1.19 |
| DST | 1.14E-07 | 3.82E-06 | -1.20 |
| IMPA2 | 2.16E-10 | 2.02E-08 | -1.20 |
| TC2N | 4.31E-09 | 2.50E-07 | -1.20 |
| CXCL12 | 1.41E-09 | 9.70E-08 | -1.20 |
| ABHD3 | 1.06E-10 | 1.12E-08 | -1.20 |
| HHIP | 4.68E-12 | 8.39E-10 | -1.20 |
| CALCOCO2 | 6.03E-11 | 7.07E-09 | -1.20 |
| MTUS1 | 7.74E-09 | 4.10E-07 | -1.20 |
| RPS6KA6 | 7.69E-07 | 1.88E-05 | -1.20 |
| LPAR5 | 1.83E-08 | 8.41E-07 | -1.20 |
| NAAA | 1.15E-10 | 1.19E-08 | -1.20 |
| P2RY14 | 3.05E-10 | 2.69E-08 | -1.21 |
| LOC100505549 | 5.93E-10 | 4.65E-08 | -1.21 |
| SLC51B | 2.12E-10 | 2.00E-08 | -1.21 |
| PVRL3 | 2.97E-06 | 5.77E-05 | -1.21 |
| FLJ36848 | 6.43E-12 | 1.10E-09 | -1.21 |
| CDS1 | 3.18E-07 | 8.88E-06 | -1.22 |
| CD24 | 5.48E-05 | 0.00063577 | -1.22 |
| XK | 2.34E-08 | 1.02E-06 | -1.22 |
| SEPP1 | 2.86E-06 | 5.59E-05 | -1.22 |
| MXD1 | 3.38E-09 | 2.05E-07 | -1.22 |
| TPSG1 | 7.98E-09 | 4.21E-07 | -1.22 |
| VIP | 8.18E-06 | 0.00013262 | -1.22 |
| RNLS | 9.49E-08 | 3.30E-06 | -1.22 |
| SPPL2A | 3.55E-10 | 3.06E-08 | -1.23 |
| EDN3 | 3.30E-11 | 4.33E-09 | -1.23 |
| FOXF2 | 4.94E-08 | 1.91E-06 | -1.23 |
| TMEM107 | 1.64E-08 | 7.67E-07 | -1.23 |
| SEMA6D | 6.25E-12 | 1.08E-09 | -1.23 |
| SMIM14 | 1.26E-13 | 4.62E-11 | -1.23 |
| CKAP2 /// IGLC1 /// IGLV1-40 /// IGLV1-40 /// IGLV1-50 /// IGLV1-50 | 2.66E-08 | 1.14E-06 | -1.23 |
| FA2H | 4.13E-07 | 1.10E-05 | -1.24 |
| HIF3A | 3.97E-08 | 1.60E-06 | -1.24 |
| FAM107B | 1.33E-08 | 6.43E-07 | -1.24 |
| STEAP4 | 5.52E-07 | 1.41E-05 | -1.24 |
| IDH3A | 5.17E-16 | 5.29E-13 | -1.24 |
| CLIC5 | 1.36E-08 | 6.56E-07 | -1.24 |
| ADAM28 | 6.65E-12 | 1.12E-09 | -1.25 |
| KBTBD11 | 6.11E-11 | 7.13E-09 | -1.25 |
| CYSTM1 | 4.13E-10 | 3.48E-08 | -1.25 |
| MEP1A | 8.99E-09 | 4.68E-07 | -1.25 |
| CMAHP | 8.42E-07 | 2.02E-05 | -1.25 |
| ABCD3 | 6.39E-10 | 4.94E-08 | -1.26 |
| SIAE | 4.53E-08 | 1.78E-06 | -1.26 |
| PRDX6 | 1.80E-10 | 1.74E-08 | -1.26 |
| SPON1 | 1.36E-09 | 9.46E-08 | -1.26 |
| SH3BGRL2 | 7.66E-08 | 2.75E-06 | -1.26 |
| USP53 | 1.09E-08 | 5.52E-07 | -1.27 |
| MFSD4 | 1.16E-09 | 8.17E-08 | -1.27 |
| BCL2L15 | 6.27E-09 | 3.42E-07 | -1.27 |
| SYNPO2 | 7.12E-06 | 0.00011824 | -1.27 |
| SLC3A1 | 9.84E-08 | 3.40E-06 | -1.28 |
| TPH1 | 4.05E-09 | 2.36E-07 | -1.28 |
| FAM162A | 5.12E-11 | 6.12E-09 | -1.28 |
| PIK3CG | 2.13E-08 | 9.51E-07 | -1.28 |
| LINC01004 | 3.85E-07 | 1.04E-05 | -1.28 |
| SLC35A3 | 2.09E-08 | 9.38E-07 | -1.29 |
| PAQR8 | 4.00E-08 | 1.61E-06 | -1.30 |
| CAPN5 | 2.08E-09 | 1.35E-07 | -1.30 |
| CA12 | 1.67E-10 | 1.64E-08 | -1.30 |
| BMP6 | 1.95E-13 | 6.59E-11 | -1.31 |
| PLCD3 | 6.91E-09 | 3.73E-07 | -1.32 |
| IGLC1 | 8.17E-07 | 1.97E-05 | -1.32 |
| C2orf88 | 1.62E-11 | 2.38E-09 | -1.32 |
| GLTP | 5.33E-12 | 9.41E-10 | -1.32 |
| GOLGA2P5 | 7.49E-10 | 5.64E-08 | -1.33 |
| CDC42EP5 | 1.90E-11 | 2.72E-09 | -1.33 |
| IL6R | 1.63E-07 | 5.16E-06 | -1.33 |
| VWA5A | 1.17E-09 | 8.26E-08 | -1.33 |
| ISX | 6.80E-10 | 5.21E-08 | -1.33 |
| ENDOD1 | 1.08E-11 | 1.68E-09 | -1.34 |
| C8orf4 | 7.40E-12 | 1.21E-09 | -1.34 |
| PAPSS2 | 5.50E-11 | 6.53E-09 | -1.34 |
| PTN | 1.06E-08 | 5.38E-07 | -1.34 |
| GFRA1 | 1.72E-07 | 5.40E-06 | -1.35 |
| ADAMTS1 | 3.52E-07 | 9.69E-06 | -1.35 |
| CTNND1 /// TMX2-CTNND1 | 4.57E-09 | 2.63E-07 | -1.35 |
| FAM46C | 1.73E-10 | 1.69E-08 | -1.35 |
| MYO5B | 3.52E-09 | 2.11E-07 | -1.35 |
| SRI | 1.99E-11 | 2.81E-09 | -1.35 |
| ZNF575 | 5.93E-13 | 1.65E-10 | -1.36 |
| UGP2 | 7.04E-10 | 5.35E-08 | -1.36 |
| EPB41L3 | 1.57E-07 | 5.02E-06 | -1.37 |
| RAPGEFL1 | 1.11E-12 | 2.62E-10 | -1.37 |
| SCIN | 9.32E-09 | 4.82E-07 | -1.38 |
| SHROOM3 | 4.90E-11 | 5.94E-09 | -1.38 |
| PANK3 | 6.78E-13 | 1.79E-10 | -1.38 |
| SEMA6A | 2.88E-11 | 3.85E-09 | -1.38 |
| LGALS2 | 3.24E-14 | 1.52E-11 | -1.38 |
| ELOVL6 | 5.77E-13 | 1.62E-10 | -1.38 |
| HAGLR | 7.83E-10 | 5.84E-08 | -1.39 |
| SLCO2A1 | 3.09E-09 | 1.90E-07 | -1.39 |
| EDNRB | 2.54E-12 | 5.10E-10 | -1.39 |
| CES3 | 3.24E-12 | 6.18E-10 | -1.39 |
| RETSAT | 2.54E-12 | 5.10E-10 | -1.39 |
| CASD1 | 3.36E-13 | 1.03E-10 | -1.40 |
| LOC100293211 | 4.11E-10 | 3.48E-08 | -1.40 |
| TPSAB1 /// TPSB2 | 1.84E-08 | 8.45E-07 | -1.40 |
| LEPREL1 | 1.73E-09 | 1.15E-07 | -1.40 |
| MTM1 | 2.22E-10 | 2.07E-08 | -1.40 |
| CD36 | 8.24E-11 | 9.05E-09 | -1.40 |
| LOC101927206 | 4.01E-11 | 5.06E-09 | -1.40 |
| PLS1 | 3.30E-09 | 2.01E-07 | -1.40 |
| ATP10B | 6.56E-08 | 2.41E-06 | -1.40 |
| SLC41A2 | 9.30E-11 | 1.00E-08 | -1.41 |
| INSL5 | 1.52E-05 | 0.00022142 | -1.41 |
| MADCAM1 | 5.21E-10 | 4.15E-08 | -1.41 |
| B3GNT7 | 1.37E-08 | 6.59E-07 | -1.42 |
| RCAN2 | 1.18E-10 | 1.22E-08 | -1.42 |
| TLCD2 | 3.62E-13 | 1.09E-10 | -1.42 |
| TST | 1.26E-09 | 8.83E-08 | -1.43 |
| IGHA1 /// IGHG1 /// IGHM /// IGHV3-23 /// IGHV4-31 | 4.02E-09 | 2.35E-07 | -1.43 |
| MPC1 | 1.41E-10 | 1.41E-08 | -1.43 |
| HAPLN1 | 5.87E-13 | 1.64E-10 | -1.43 |
| PBLD | 1.34E-06 | 2.98E-05 | -1.43 |
| MIR4680 /// PDCD4 | 1.83E-15 | 1.56E-12 | -1.44 |
| VSIG2 | 8.29E-12 | 1.33E-09 | -1.44 |
| HSPB6 | 2.01E-06 | 4.17E-05 | -1.44 |
| CCL28 | 2.19E-11 | 3.06E-09 | -1.44 |
| RP4-714D9.5 | 5.91E-14 | 2.50E-11 | -1.44 |
| S100A10 | 3.42E-11 | 4.46E-09 | -1.45 |
| GTF2A2 | 5.65E-09 | 3.16E-07 | -1.45 |
| C4orf19 | 9.43E-11 | 1.01E-08 | -1.45 |
| CDH17 | 2.06E-07 | 6.22E-06 | -1.45 |
| HIGD1A | 8.07E-13 | 2.06E-10 | -1.46 |
| FOXD2 | 1.32E-09 | 9.20E-08 | -1.46 |
| BBIP1 | 1.96E-15 | 1.61E-12 | -1.47 |
| IGKC /// IGKV1-13 /// IGKV1-13 | 2.40E-10 | 2.21E-08 | -1.48 |
| RASSF6 | 1.16E-08 | 5.77E-07 | -1.48 |
| SLC44A4 | 1.23E-10 | 1.25E-08 | -1.48 |
| FUCA1 | 4.79E-13 | 1.37E-10 | -1.49 |
| CCL8 | 6.73E-06 | 0.00011295 | -1.50 |
| ATP8B1 | 4.05E-12 | 7.49E-10 | -1.50 |
| ZBTB7C | 3.18E-13 | 9.85E-11 | -1.50 |
| CYAT1 /// IGLV1-44 | 4.24E-11 | 5.30E-09 | -1.50 |
| GCSAM | 5.21E-11 | 6.21E-09 | -1.50 |
| TP53INP2 | 7.95E-13 | 2.04E-10 | -1.50 |
| MIR22 /// MIR22HG | 1.19E-13 | 4.39E-11 | -1.51 |
| IGHD /// IGHG1 /// IGHM | 6.50E-09 | 3.54E-07 | -1.51 |
| CDHR2 | 4.96E-09 | 2.83E-07 | -1.52 |
| C1orf21 | 6.82E-11 | 7.76E-09 | -1.52 |
| CMBL | 7.95E-08 | 2.84E-06 | -1.52 |
| EPB41L4B | 9.76E-09 | 5.02E-07 | -1.53 |
| SLC35D1 | 1.67E-12 | 3.64E-10 | -1.53 |
| GPD1L | 2.08E-11 | 2.92E-09 | -1.54 |
| HPGD | 1.14E-10 | 1.18E-08 | -1.54 |
| THBS1 | 2.73E-10 | 2.46E-08 | -1.54 |
| SCARA5 | 4.99E-12 | 8.92E-10 | -1.56 |
| AGR2 | 1.68E-07 | 5.29E-06 | -1.57 |
| TBCAP2 /// TBCAP2 | 1.09E-11 | 1.68E-09 | -1.58 |
| ADTRP | 2.00E-13 | 6.72E-11 | -1.59 |
| LINC01133 | 7.66E-08 | 2.75E-06 | -1.59 |
| FRMD3 | 4.62E-11 | 5.67E-09 | -1.59 |
| EPB41L4A-AS1 | 1.97E-12 | 4.19E-10 | -1.60 |
| CA4 | 3.00E-10 | 2.66E-08 | -1.61 |
| CES2 | 5.28E-08 | 2.02E-06 | -1.61 |
| TMPRSS2 | 1.15E-12 | 2.68E-10 | -1.61 |
| IGKV1OR2-108 /// IGKV1OR2-108 | 6.72E-10 | 5.16E-08 | -1.62 |
| SLAMF7 | 8.65E-08 | 3.04E-06 | -1.64 |
| IRF4 | 1.27E-10 | 1.28E-08 | -1.64 |
| MAMDC2 | 1.18E-10 | 1.22E-08 | -1.65 |
| TRPA1 | 5.92E-12 | 1.04E-09 | -1.65 |
| TMEM30B | 2.41E-11 | 3.31E-09 | -1.65 |
| BMS1P20 | 7.48E-09 | 3.98E-07 | -1.66 |
| GUCA2B | 1.49E-08 | 7.09E-07 | -1.66 |
| BTNL8 | 2.32E-10 | 2.14E-08 | -1.67 |
| ANPEP | 9.96E-07 | 2.33E-05 | -1.67 |
| F2RL1 | 3.84E-11 | 4.87E-09 | -1.67 |
| CYP4F12 | 2.44E-09 | 1.55E-07 | -1.68 |
| SQRDL | 2.70E-16 | 3.15E-13 | -1.68 |
| B3GALT5 | 1.08E-10 | 1.13E-08 | -1.68 |
| SLC4A4 | 2.83E-11 | 3.80E-09 | -1.68 |
| MUC12 | 1.28E-08 | 6.25E-07 | -1.69 |
| A1CF | 3.07E-12 | 5.91E-10 | -1.69 |
| BCAS1 | 1.86E-15 | 1.57E-12 | -1.69 |
| NAT2 | 7.20E-11 | 8.08E-09 | -1.69 |
| MOGAT2 | 7.97E-11 | 8.79E-09 | -1.69 |
| SLC30A10 | 4.68E-08 | 1.83E-06 | -1.69 |
| NRG1 | 1.88E-11 | 2.71E-09 | -1.71 |
| PYY | 2.80E-10 | 2.51E-08 | -1.71 |
| IGHG1 | 1.18E-08 | 5.84E-07 | -1.72 |
| FOSB | 2.60E-08 | 1.12E-06 | -1.73 |
| SOX10 | 1.47E-13 | 5.14E-11 | -1.73 |
| PIGZ | 1.31E-10 | 1.32E-08 | -1.73 |
| ABCG2 | 1.54E-06 | 3.33E-05 | -1.73 |
| CKMT1A /// CKMT1B | 7.83E-11 | 8.68E-09 | -1.74 |
| IGHM | 6.59E-05 | 0.00073943 | -1.75 |
| FHL1 | 2.81E-12 | 5.53E-10 | -1.78 |
| AGPAT9 | 4.09E-12 | 7.53E-10 | -1.78 |
| RETNLB | 6.05E-07 | 1.53E-05 | -1.79 |
| ADAM22 | 2.24E-11 | 3.12E-09 | -1.80 |
| HK2 | 2.89E-11 | 3.85E-09 | -1.80 |
| LOC101928620 /// POU2AF1 | 9.61E-12 | 1.52E-09 | -1.80 |
| ABCA8 | 2.75E-12 | 5.47E-10 | -1.81 |
| FXYD3 | 4.93E-10 | 3.99E-08 | -1.81 |
| SATB2 | 2.74E-08 | 1.17E-06 | -1.81 |
| ANO7 | 2.41E-11 | 3.31E-09 | -1.81 |
| LEFTY1 | 1.11E-05 | 0.00017102 | -1.81 |
| SMPDL3A | 3.80E-13 | 1.13E-10 | -1.81 |
| IGLV1-44 | 1.31E-11 | 1.97E-09 | -1.82 |
| CLDN23 | 1.62E-13 | 5.57E-11 | -1.82 |
| IGKC /// IGKV1-39 /// IGKV1-39 /// IGKV1D-39 /// IGKV1D-39 | 6.30E-08 | 2.33E-06 | -1.83 |
| DNASE1L3 | 5.01E-11 | 6.02E-09 | -1.84 |
| NKX2-3 | 9.43E-12 | 1.49E-09 | -1.84 |
| SLC9A2 | 4.61E-13 | 1.33E-10 | -1.84 |
| AOC1 | 4.03E-12 | 7.49E-10 | -1.84 |
| MUC3 | 1.97E-08 | 8.90E-07 | -1.84 |
| IGKV1-17 /// IGKV1-17 | 1.55E-09 | 1.05E-07 | -1.85 |
| EFNA2 | 1.47E-10 | 1.46E-08 | -1.85 |
| SEMA4G | 5.92E-14 | 2.50E-11 | -1.86 |
| CKB | 6.08E-13 | 1.67E-10 | -1.87 |
| IGKC /// IGKV2-28 /// IGKV2-28 /// IGKV2D-28 /// IGKV2D-28 | 6.97E-12 | 1.16E-09 | -1.88 |
| IQGAP2 | 1.52E-15 | 1.40E-12 | -1.88 |
| METTL7A | 1.73E-10 | 1.69E-08 | -1.88 |
| TDP2 | 1.00E-11 | 1.57E-09 | -1.89 |
| abParts /// IGKC /// IGKV4-1 /// IGKV4-1 | 7.65E-09 | 4.06E-07 | -1.90 |
| MDM2 | 5.42E-14 | 2.34E-11 | -1.91 |
| BBIP1 /// LOC100130175 | 2.72E-12 | 5.42E-10 | -1.91 |
| ETHE1 | 2.61E-12 | 5.21E-10 | -1.91 |
| MT1E | 3.81E-11 | 4.85E-09 | -1.92 |
| KLHL6 | 1.98E-08 | 8.95E-07 | -1.92 |
| IGHA1 /// IGHV4-31 | 1.65E-10 | 1.62E-08 | -1.92 |
| DSC2 | 2.15E-12 | 4.42E-10 | -1.93 |
| SATB2-AS1 | 1.38E-09 | 9.54E-08 | -1.93 |
| LOC100652777 /// PLA2G10 | 2.00E-11 | 2.81E-09 | -1.94 |
| IGHG1 /// IGHV4-59 /// IGHV4-59 /// IGHV4-61 /// IGHV4-61 | 4.62E-10 | 3.80E-08 | -1.94 |
| AKR1B10 | 5.67E-11 | 6.70E-09 | -1.95 |
| AGR3 | 3.41E-08 | 1.40E-06 | -1.98 |
| ARHGAP44 | 9.89E-13 | 2.46E-10 | -1.98 |
| LRRC19 | 2.15E-12 | 4.42E-10 | -1.98 |
| IGHV4-31 | 3.21E-10 | 2.80E-08 | -1.99 |
| CTD-2325A15.5 | 2.06E-10 | 1.96E-08 | -1.99 |
| CEACAM1 | 2.50E-12 | 5.08E-10 | -2.00 |
| LGALS4 | 1.12E-13 | 4.26E-11 | -2.00 |
| CCL15 /// CCL15-CCL14 | 2.58E-10 | 2.35E-08 | -2.00 |
| SGK1 | 7.77E-13 | 2.00E-10 | -2.01 |
| SCNN1G | 7.25E-08 | 2.63E-06 | -2.01 |
| HOXD3 /// HOXD4 /// LOC401021 | 3.91E-17 | 9.22E-14 | -2.01 |
| UGDH | 5.14E-17 | 1.13E-13 | -2.02 |
| HSD17B2 | 3.39E-13 | 1.04E-10 | -2.03 |
| IGLL5 | 1.25E-10 | 1.27E-08 | -2.05 |
| ST6GALNAC1 | 3.42E-13 | 1.04E-10 | -2.07 |
| RP11-38P22.2 | 2.06E-14 | 1.10E-11 | -2.08 |
| CWH43 | 6.93E-13 | 1.82E-10 | -2.08 |
| IGHD | 1.59E-09 | 1.07E-07 | -2.09 |
| IGLC1 /// IGLJ3 /// IGLV2-14 /// IGLV2-23 /// IGLV2-23 /// IGLV@ | 2.09E-09 | 1.36E-07 | -2.10 |
| PARM1 | 3.03E-14 | 1.45E-11 | -2.11 |
| CFD | 2.87E-17 | 7.87E-14 | -2.11 |
| MAOA | 1.64E-12 | 3.61E-10 | -2.13 |
| PIGR | 4.04E-08 | 1.62E-06 | -2.15 |
| GCNT3 | 4.68E-11 | 5.73E-09 | -2.16 |
| MALL | 6.26E-14 | 2.60E-11 | -2.20 |
| VIPR1 | 1.80E-15 | 1.56E-12 | -2.20 |
| LYPD8 | 1.75E-10 | 1.71E-08 | -2.22 |
| SPINK5 | 9.21E-11 | 9.97E-09 | -2.22 |
| ITLN1 | 6.47E-11 | 7.43E-09 | -2.24 |
| BMP2 | 1.44E-13 | 5.06E-11 | -2.24 |
| MT1M | 4.58E-10 | 3.78E-08 | -2.24 |
| LOC101928284 | 3.70E-12 | 6.94E-10 | -2.24 |
| ARL14 | 1.67E-12 | 3.64E-10 | -2.24 |
| CD79A | 9.21E-12 | 1.46E-09 | -2.24 |
| CA7 | 1.77E-14 | 9.78E-12 | -2.25 |
| FGFBP1 | 1.37E-11 | 2.06E-09 | -2.28 |
| IGKC /// IGKV2-28 /// IGKV2-28 /// IGKV2D-28 /// IGKV2D-28 /// IGKV2OR22-4 /// IGKV2OR22-4 | 5.08E-12 | 9.01E-10 | -2.29 |
| ITM2C | 4.11E-17 | 9.34E-14 | -2.30 |
| AQP8 | 2.18E-08 | 9.68E-07 | -2.31 |
| NR3C2 | 3.00E-18 | 1.23E-14 | -2.32 |
| AHCYL2 | 4.75E-15 | 3.32E-12 | -2.35 |
| AK057259 /// AK096803 | 3.10E-10 | 2.73E-08 | -2.35 |
| TSPAN1 | 1.19E-14 | 6.95E-12 | -2.35 |
| IGH /// IGHA2 | 5.11E-10 | 4.10E-08 | -2.39 |
| C15orf48 | 6.50E-15 | 4.11E-12 | -2.40 |
| PDE9A | 1.29E-12 | 2.94E-10 | -2.41 |
| SULT1B1 | 2.32E-15 | 1.88E-12 | -2.42 |
| AL928768.3 /// Ig alpha 1-[alpha]2m /// IGH | 1.91E-14 | 1.03E-11 | -2.44 |
| NXPE1 | 1.50E-16 | 2.07E-13 | -2.50 |
| SELENBP1 | 1.57E-15 | 1.42E-12 | -2.52 |
| BTNL3 | 1.71E-14 | 9.51E-12 | -2.54 |
| ADH1C | 1.29E-12 | 2.94E-10 | -2.57 |
| PLA2G2A | 7.26E-09 | 3.88E-07 | -2.57 |
| SLC17A4 | 1.20E-12 | 2.76E-10 | -2.61 |
| KLF4 | 5.52E-15 | 3.68E-12 | -2.62 |
| CCL15 | 2.66E-13 | 8.55E-11 | -2.62 |
| MUC5B | 1.13E-07 | 3.80E-06 | -2.63 |
| UGT1A1 /// UGT1A3 /// UGT1A5 /// UGT1A8 /// UGT1A9 | 1.84E-11 | 2.67E-09 | -2.65 |
| GPA33 | 3.32E-13 | 1.02E-10 | -2.65 |
| HMGCS2 | 1.57E-09 | 1.07E-07 | -2.66 |
| FKBP1A-SDCBP2 /// SDCBP2 | 4.45E-15 | 3.16E-12 | -2.67 |
| MT1H | 2.15E-10 | 2.02E-08 | -2.67 |
| MUC2 | 3.43E-09 | 2.08E-07 | -2.75 |
| TSPAN7 | 1.91E-16 | 2.49E-13 | -2.75 |
| LOC340184 | 3.94E-16 | 4.24E-13 | -2.79 |
| GCG | 2.46E-10 | 2.25E-08 | -2.94 |
| DHRS11 | 1.32E-16 | 1.95E-13 | -2.95 |
| MUC4 | 3.91E-11 | 4.94E-09 | -2.96 |
| CHP2 | 2.58E-15 | 1.98E-12 | -3.04 |
| CLDN8 | 4.35E-12 | 7.95E-10 | -3.07 |
| PKIB | 2.64E-17 | 7.72E-14 | -3.12 |
| C10orf99 | 2.17E-16 | 2.71E-13 | -3.14 |
| PLAC8 | 2.89E-12 | 5.65E-10 | -3.15 |
| ABCC13 | 1.03E-15 | 9.59E-13 | -3.21 |
| HSD11B2 | 3.21E-17 | 7.87E-14 | -3.25 |
| SLC26A2 | 8.49E-15 | 5.16E-12 | -3.25 |
| SST | 9.84E-13 | 2.45E-10 | -3.27 |
| FABP1 | 1.06E-12 | 2.54E-10 | -3.30 |
| FCGBP | 1.12E-13 | 4.26E-11 | -3.33 |
| IGH /// IGHA1 /// IGHA2 | 4.96E-15 | 3.38E-12 | -3.37 |
| FABP1 /// PRDM10 | 1.91E-11 | 2.73E-09 | -3.37 |
| ADH1B | 2.51E-19 | 2.60E-15 | -3.40 |
| PCK1 | 2.63E-14 | 1.32E-11 | -3.43 |
| CD177 | 7.73E-13 | 2.00E-10 | -3.47 |
| IGJ | 6.00E-14 | 2.52E-11 | -3.47 |
| NXPE4 | 3.18E-17 | 7.87E-14 | -3.51 |
| DHRS9 | 9.41E-14 | 3.68E-11 | -3.51 |
| KRT20 | 1.47E-14 | 8.36E-12 | -3.54 |
| PADI2 | 8.03E-16 | 7.95E-13 | -3.60 |
| CEACAM7 | 1.02E-15 | 9.59E-13 | -3.67 |
| UGT2B17 | 2.33E-10 | 2.15E-08 | -3.71 |
| GUCA2A | 5.31E-15 | 3.58E-12 | -3.75 |
| ADAMDEC1 | 8.72E-18 | 2.97E-14 | -3.89 |
| ZG16 | 1.66E-16 | 2.21E-13 | -4.05 |
| SCNN1B | 6.54E-19 | 4.46E-15 | -4.11 |
| CA1 | 2.54E-19 | 2.60E-15 | -4.32 |
| CHGA | 5.16E-20 | 1.05E-15 | -4.34 |
| MS4A12 | 3.21E-19 | 2.81E-15 | -5.01 |
| CLCA4 | 2.01E-16 | 2.57E-13 | -5.07 |
| CA2 | 1.36E-20 | 4.17E-16 | -5.11 |
| CLCA1 | 6.41E-17 | 1.16E-13 | -5.21 |
| SLC26A3 | 1.75E-18 | 8.28E-15 | -5.30 |

**Supplementary Table 5**. Number of differentially expressed genes in the integrated mRNA microarray analysis

| **Accesion ID** | **DEG** | **Up-Regulated mRNAs** | **Down-Regulated mRNAs** |
| --- | --- | --- | --- |
| **GSE9348** | 4874 | 2188 | 2686 |
| **GSE21510** | 5634 | 3207 | 2427 |
| **GSE24550** | 7858 | 7735 | 123 |

**Supplementary Table 6**. Number of differentially expressed genes in the miRNA microarray dataset analysis

| **Datasets** | **DEMs** | **Up-Regulated miRNAs** | **Down-Regulated miRNAs** |
| --- | --- | --- | --- |
| Affymetrix E-MTAB-752,  E-GEOD-35834 and GSE68377 | 110 | 63 | 47 |
| Agilent GSE35982, E-MTAB-813 datasets | 90 | 75 | 15 |
|  |  |  |  |

**Supplementary Table 7**. Number common genes obtained by intersecting the miRNA target genes and the DEGs from our discovery dataset

| **miRNA** | **Affymetrix** | **Agilent** | **Gene symbol** | **Target gene fold change in discovery dataset** | **Pathways** |
| --- | --- | --- | --- | --- | --- |
| hsa-miR-139-5p | -6.88 | -2,825 | HSP90AA1 | 2.02905 | [Estrogen signaling pathway](https://david.ncifcrf.gov/kegg.jsp?path=hsa04915$Estrogen%20signaling%20pathway&termId=550028791&source=kegg) |
| hsa-mir-497 | -3.565 | -1.78 | HSP90AA1 | 2.02905 | [pathway in cancer, PI3K-Akt signaling pathway](https://david.ncifcrf.gov/kegg.jsp?path=hsa04915$Estrogen%20signaling%20pathway&termId=550028791&source=kegg) |
|  | -3.565 | -1.78 | CCND1 | 2.34638 | pathway in cancer, PI3K-Akt signaling pathway, Endometrial cancer, Colorectal cancer, Bladder cancer |
|  | -3.565 | -1.78 | VEGFA | 2.96671 | pathway in cancer, PI3K-Akt signaling pathway, Bladder cancer |
|  | -3.565 | -1.78 | AXIN2 | 2.35901 | pathway in cancer, Endometrial cancer, Colorectal cancer |
| hsa-mir-378 | -3.061 | -1,609 | F2R | 2.01037 | Pathways in cancer, PI3K-Akt signaling pathway |
|  |  |  | HSP90AA1 | 2.02905 | Pathways in cancer, PI3K-Akt signaling pathway |
|  |  |  | VEGFA | 2.96671 | Pathways in cancer, Renal cell carcinoma, PI3K-Akt signaling pathway |
|  |  |  | TGFB2 | 2.11608 | Pathways in cancer, Renal cell carcinoma |
| hsa-mir-195 | -3.047 | -2.41 | CCND1 | 2.34638 | pathway in cancer, Endometrial cancer, Colorectal cancer, Bladder cancer |
|  |  |  | VEGFA | 2.96671 | pathway in cancer, Bladder cancer |
|  |  |  | AXIN2 | 2.35901 | pathway in cancer, Endometrial cancer, Colorectal cancer |
| hsa-mir-145 | -2.868 | -2,084 | CCND1 | 2.34638 | Pathways in cancer, Proteoglycans in cancer, p53 signaling pathway, MicroRNAs in cancer, PI3K-Akt signaling pathway, FoxO signaling pathway,Focal adhesion |
|  |  |  | VEGFA | 2.96671 | Pathways in cancer, Proteoglycans in cancer, MicroRNAs in cancer, PI3K-Akt signaling pathway, Focal adhesion |
|  |  |  | MMP1 | 4.31942 | Pathways in cancer |
|  |  |  | STAT1 | 2.25168 | Pathways in cancer |
|  |  |  | CD44 | 2.04554 | Proteoglycans in cancer, MicroRNAs in cancer |
|  |  |  | SERPINE1 | 3.62393 | p53 signaling pathway |
|  |  |  | LOC100129518 /// SOD2 | 2.04265 | FoxO signaling pathway |
|  |  |  | COL5A1 | 3.74258 | PI3K-Akt signaling pathway, Focal adhesion |
| hsa-mir-150 | -2.8 | -2,356 | VEGFA | 2.96671 | Proteoglycans in cancer, Ras signaling pathway, MicroRNAs in cancer |
|  |  |  | ELK1 | 2.04755 | Proteoglycans in cancer, Ras signaling pathway |
|  |  |  | PLA2G16 | 2.20188 | Ras signaling pathway, Arachidonic acid metabolism |
|  |  |  | NOTCH3 | 2.81924 | MicroRNAs in cancer |
|  |  |  | PTGIS | 2.12231 | Arachidonic acid metabolism |
| hsa-mir-133a | -2.799 | -1,794 | VEGFA | 2.96671 | PI3K-Akt signaling pathway, Focal adhesion, |
|  |  |  | THBS2 | 20.2049 | PI3K-Akt signaling pathway, Focal adhesion, ECM-receptor interaction |
|  |  |  | COL1A1 | 7.46996 | PI3K-Akt signaling pathway, Focal adhesion, ECM-receptor interaction |
|  |  |  | BCL2L1 | 2.26022 | PI3K-Akt signaling pathway, ECM-receptor interaction |
| hsa-mir-375 | -2.686 | -1,949 | NA |  |  |
| hsa-mir-10b | -2.616 | -1,563 | NA |  |  |
| hsa-mir-30a | -2.256 | -2,038 | WNT5A | 2.23228 | Proteoglycans in cancer |
|  |  |  | CD44 | 2.04554 | Proteoglycans in cancer |
|  |  |  | MET | 2.35032 | Proteoglycans in cancer, Axon guidance |
|  |  |  | PLXNA1 | 2.04482 | Axon guidance |
| hsa-mir-133b | -2.046 | -2,359 | NA |  |  |
| hsa-mir-342-5p | -1.874 | -1,989 | NA |  |  |
| hsa-mir-1 | -1.554 | -2,559 | CD44 | 2.04554 | Proteoglycans in cancer, MicroRNAs in cancer, ECM-receptor interaction |
|  |  |  | CCND1 | 2.34638 | Proteoglycans in cancer, MicroRNAs in cancer, Focal adhesion, PI3K-Akt signaling pathway |
|  |  |  | MET | 2.35032 | Proteoglycans in cancer, MicroRNAs in cancer, Focal adhesion, PI3K-Akt signaling pathway |
|  |  |  | FN1 | 2.70179 | Proteoglycans in cancer, Focal adhesion, PI3K-Akt signaling pathway, ECM-receptor interaction |
|  |  |  | TIMP3 | 2.67179 | Proteoglycans in cancer, MicroRNAs in cancer |
| hsa-mir-93 | 1.32 | 2,365 | BMP2 | -4.74775 | Pathways in cancer |
|  |  |  | MDM2 | -3.75246 | Pathways in cancer, Proteoglycans in cancer, PI3K-Akt signaling pathway |
|  |  |  | PRKACB | -4.07373 | Pathways in cancer, Proteoglycans in cancer, Wnt signaling pathway |
|  |  |  | TCF7L2 | -2.03584 | Pathways in cancer, Wnt signaling pathway |
|  |  |  | WNT2B | -2.08947 | Pathways in cancer, Proteoglycans in cancer, Wnt signaling pathway |
| hsa-mir-27a | 1.324 | 2,141 | PIK3CG | -2.55314 | Pathways in cancer, Proteoglycans in cancer, Proteoglycans in cancer, Colorectal cancer, PI3K-Akt signaling pathway |
| hsa-mir-1280 | 1.525 | 1,643 | NA |  |  |
| hsa-mir-148a | 1.536 | 2,123 | WNT2B | -2.08947 | Pathways in cancer |
| hsa-mir-92b | 1.536 | 2,258 | HMGCR | -2.12813 | Biosynthesis of antibiotics |
| hsa-mir-331-3p | 1.555 | 1,593 | PPARD | -2.02996 | PPAR signaling pathway |
| hsa-mir-29a | 1.608 | 1,958 | SGK1 | -4.02026 | PI3K-Akt signaling pathway, FoxO signaling pathway |
|  |  |  | MDM2 | -4.02846 | PI3K-Akt signaling pathway, Pathways in cancer, MicroRNAs in cancer |
|  |  |  | FOS | -2.10065 | Pathways in cancer |
| hsa-mir-29b | 1.653 | 2,314 | PIK3CG | -2.55314 |  |
|  |  |  | SGK1 | -4.02026 | PI3K-Akt signaling pathway |
|  |  |  | MDM2 | -4.02846 | PI3K-Akt signaling pathway, Pathways in cancer, MicroRNAs in cancer |
|  |  |  | FOS | -2.10065 | Pathways in cancer, Colorectal cancer |
|  |  |  | PPARD | -2.02996 | Pathways in cancer |
| hsa-mir-92a | 1.653 | 2,505 | MDM2 | -4.02846 | Pathways in cancer, PI3K-Akt signaling pathway, p53 signaling pathway |
|  |  |  | PPARD | -2.02996 | Pathways in cancer, Wnt signaling pathway |
| hsa-mir-106b | 1.679 | 2,179 | TCF7L2 | -2.03584 | Pathways in cancer, Hippo signaling pathway, Wnt signaling pathway |
|  |  |  | PRKACB | -2.13471 | Proteoglycans in cancer, Pathways in cancer, Wnt signaling pathway |
|  |  |  | MDM2 | -4.02846 | Proteoglycans in cancer, Pathways in cancer |
|  |  |  | BMP2 | -4.72228 | Pathways in cancer, Hippo signaling pathway |
| hsa-mir-34a | 1.679 | 2,803 | NA |  |  |
| hsa-mir-20b | 1.72 | 2,447 | TCF7L2 | -2.03584 | wnt signaling pathway |
|  |  |  | PRKACB | -2.13471 | wnt signaling pathway |
|  |  |  | MDM2 | -4.02846 | FoxO signaling pathway |
| hsa-mir-19b | 1.756 | 2,449 | FGFR2 | -2.21463 | MAPK signaling pathway |
|  |  |  | FOS | -2.10065 | MAPK signaling pathway |
|  |  |  | CACNB2 | -2.12154 | MAPK signaling pathway |
|  |  |  | PRKACB | -2.13471 | MAPK signaling pathway |
| hsa-mir-130b | 1.771 | 1,994 | HHIP | -2.2979 | Hedgehog signaling pathway, Basal cell carcinoma |
|  |  |  | TCF7L2 | -2.03584 | Basal cell carcinoma |
| hsa-mir-17 | 1.84 | 2,709 | PRKACB | -2.13471 | Proteoglycans in cancer, Pathways in cancer, Wnt signaling pathway |
|  |  |  | THBS1 | -2.9164 | Proteoglycans in cancer, PI3K-Akt signaling pathway, MicroRNAs in cancer |
|  |  |  | BMP2 | -4.72228 | Pathways in cancer |
|  |  |  | MDM2 | -4.02846 | Proteoglycans in cancer, Pathways in cancer, MicroRNAs in cancer, p53 signaling pathway |
|  |  |  | TCF7L2 | -2.03584 | Pathways in cancer, Wnt signaling pathway |
| hsa-mir-25 | 1.875 | 1,892 | MDM2 | -4.02846 | Prostate cancer |
| hsa-mir-203 | 1.89 | 2,356 | NA |  |  |
| hsa-mir-196b | 1.937 | 1,725 | NA |  |  |
| hsa-mir-424 | 1.946 | 3,406 | NA |  |  |
| hsa-mir-20a | 2 | 2,845 | BMP2 | -4.72228 | Pathways in cancer |
|  |  |  | MDM2 | -4.02846 | Pathways in cancer, p53 signaling pathway, Wnt signaling pathway |
|  |  |  | TCF7L2 | -2.03584 | Pathways in cancer |
|  |  |  | PRKACB | -2.13471 | Wnt signaling pathway |
| hsa-mir-1290 | 2.036 | 2,416 | NA |  |  |
| hsa-mir-552 | 2.049 | 3,157 | NA |  |  |
| hsa-mir-720 | 2.106 | 1,743 | NA |  |  |
| hsa-mir-19a | 2.156 | 2,156 | PRKACB | -2.13471 | MAPK signaling pathway |
|  |  |  | FOS | -2.10065 | MAPK signaling pathway |
|  |  |  | CACNB2 | -2.12154 | MAPK signaling pathway |
|  |  |  | THBS1 | -2.9164 | p53 signaling pathway |
| hsa-mir-18b | 2.878 | 2,215 | MDM2 | -4.02846 | p53 signaling pathway, cell cycle |
| hsa-mir-224 | 2.929 | 4,733 | MDM2 | -4.02846 | Pathways in cancer, p53 signaling pathway |
| hsa-mir-21 | 2.934 | 3,904 | MIR4680 /// PDCD4 | -2.70497 | Proteoglycans in cancer, MicroRNAs in cancer |
|  |  |  | SGK1 | -4.02026 | FoxO signaling pathway |
| hsa-mir-183 | 3.944 | 3,952 | MIR4680 /// PDCD4 | -2.70497 | Proteoglycans in cancer |
| hsa-mir-182 |  | 4.812 | MIR4680 /// PDCD4 | -2.70497 | Proteoglycans in cancer, MicroRNAs in cancer |
|  |  |  | THBS1 | -2.9164 | Proteoglycans in cancer, MicroRNAs in cancer |

**Supplementary Table 8.** GSEA results of KEGG pathway enrichment in CRC patients with high THBS2 expression in the GSE17536 dataset

| **NAME** | **SIZE** | **ES** | **NES** | **NOM p-val** | **FDR q-val** |
| --- | --- | --- | --- | --- | --- |
| KEGG_CELL_ADHESION_MOLECULES_CAMS | 125 | 0.65 | 1.87 | 0.00 | 0.02 |
| KEGG_FOCAL_ADHESION | 193 | 0.70 | 1.98 | 0.00 | 0.02 |
| KEGG_GLYCOSAMINOGLYCAN_BIOSYNTHESIS_CHONDROITIN_SULFATE | 22 | 0.76 | 1.85 | 0.00 | 0.02 |
| KEGG_ECM_RECEPTOR_INTERACTION | 81 | 0.80 | 1.93 | 0.00 | 0.02 |
| KEGG_MTOR_SIGNALING_PATHWAY | 50 | 0.57 | 1.83 | 0.00 | 0.02 |
| KEGG_DILATED_CARDIOMYOPATHY | 88 | 0.60 | 1.87 | 0.00 | 0.02 |
| KEGG_LYSOSOME | 116 | 0.59 | 1.80 | 0.00 | 0.02 |
| KEGG_GLIOMA | 63 | 0.58 | 1.81 | 0.00 | 0.02 |
| KEGG_LEUKOCYTE_TRANSENDOTHELIAL_MIGRATION | 111 | 0.62 | 1.76 | 0.00 | 0.02 |
| KEGG_AXON_GUIDANCE | 128 | 0.52 | 1.76 | 0.00 | 0.02 |
| KEGG_REGULATION_OF_ACTIN_CYTOSKELETON | 207 | 0.53 | 1.88 | 0.00 | 0.03 |
| KEGG_GAP_JUNCTION | 84 | 0.58 | 1.77 | 0.00 | 0.03 |
| KEGG_HYPERTROPHIC_CARDIOMYOPATHY_HCM | 81 | 0.55 | 1.77 | 0.00 | 0.03 |
| KEGG_VASCULAR_SMOOTH_MUSCLE_CONTRACTION | 103 | 0.56 | 1.74 | 0.00 | 0.03 |
| KEGG_EPITHELIAL_CELL_SIGNALING_IN_HELICOBACTER_PYLORI_INFECTION | 67 | 0.54 | 1.77 | 0.00 | 0.03 |
| KEGG_FC_GAMMA_R_MEDIATED_PHAGOCYTOSIS | 90 | 0.58 | 1.74 | 0.00 | 0.03 |
| KEGG_ACUTE_MYELOID_LEUKEMIA | 56 | 0.55 | 1.70 | 0.00 | 0.03 |
| KEGG_VIRAL_MYOCARDITIS | 65 | 0.68 | 1.73 | 0.00 | 0.03 |
| KEGG_PATHWAYS_IN_CANCER | 318 | 0.50 | 1.70 | 0.00 | 0.03 |
| KEGG_MELANOMA | 71 | 0.58 | 1.72 | 0.00 | 0.03 |
| KEGG_TOLL_LIKE_RECEPTOR_SIGNALING_PATHWAY | 97 | 0.64 | 1.70 | 0.00 | 0.03 |
| KEGG_SMALL_CELL_LUNG_CANCER | 84 | 0.57 | 1.71 | 0.00 | 0.03 |
| KEGG_SYSTEMIC_LUPUS_ERYTHEMATOSUS | 50 | 0.77 | 1.71 | 0.00 | 0.03 |
| KEGG_ARRHYTHMOGENIC_RIGHT_VENTRICULAR_CARDIOMYOPATHY_ARVC | 72 | 0.55 | 1.69 | 0.00 | 0.03 |
| KEGG_PROSTATE_CANCER | 89 | 0.52 | 1.64 | 0.01 | 0.05 |

.

**Supplementary Table 9.** GSEA results of KEGG pathway enrichment in CRC patients with high THBS2 expression in the GSE39582 dataset

| **NAME** | **SIZE** | **ES** | **NES** | **NOM p-val** | **FDR q-val** |
| --- | --- | --- | --- | --- | --- |
| KEGG_FOCAL_ADHESION | 193 | 0.74 | 2.23 | 0.00 | 0.00 |
| KEGG_ECM_RECEPTOR_INTERACTION | 81 | 0.85 | 2.07 | 0.00 | 0.01 |
| KEGG_AXON_GUIDANCE | 128 | 0.57 | 2.06 | 0.00 | 0.00 |
| KEGG_MTOR_SIGNALING_PATHWAY | 50 | 0.55 | 2.00 | 0.00 | 0.00 |
| KEGG_GLYCOSAMINOGLYCAN_BIOSYNTHESIS_CHONDROITIN_SULFATE | 22 | 0.83 | 1.98 | 0.00 | 0.01 |
| KEGG_SMALL_CELL_LUNG_CANCER | 84 | 0.57 | 1.97 | 0.00 | 0.01 |
| KEGG_HYPERTROPHIC_CARDIOMYOPATHY_HCM | 81 | 0.62 | 1.96 | 0.00 | 0.01 |
| KEGG_PATHWAYS_IN_CANCER | 318 | 0.52 | 1.95 | 0.00 | 0.01 |
| KEGG_REGULATION_OF_ACTIN_CYTOSKELETON | 207 | 0.52 | 1.93 | 0.00 | 0.01 |
| KEGG_DILATED_CARDIOMYOPATHY | 88 | 0.61 | 1.91 | 0.00 | 0.01 |
| KEGG_ARRHYTHMOGENIC_RIGHT_VENTRICULAR_CARDIOMYOPATHY_ARVC | 72 | 0.59 | 1.88 | 0.00 | 0.01 |
| KEGG_GLIOMA | 63 | 0.55 | 1.88 | 0.00 | 0.01 |
| KEGG_WNT_SIGNALING_PATHWAY | 146 | 0.53 | 1.87 | 0.00 | 0.01 |
| KEGG_CELL_ADHESION_MOLECULES_CAMS | 125 | 0.69 | 1.87 | 0.00 | 0.01 |
| KEGG_MELANOMA | 71 | 0.60 | 1.85 | 0.00 | 0.01 |
| KEGG_LEUKOCYTE_TRANSENDOTHELIAL_MIGRATION | 111 | 0.64 | 1.85 | 0.00 | 0.01 |
| KEGG_TOLL_LIKE_RECEPTOR_SIGNALING_PATHWAY | 97 | 0.66 | 1.84 | 0.00 | 0.01 |
| KEGG_LEISHMANIA_INFECTION | 67 | 0.74 | 1.82 | 0.00 | 0.01 |
| KEGG_ANTIGEN_PROCESSING_AND_PRESENTATION | 74 | 0.63 | 1.81 | 0.00 | 0.01 |
| KEGG_VIRAL_MYOCARDITIS | 65 | 0.69 | 1.80 | 0.00 | 0.01 |
| KEGG_GLYCOSAMINOGLYCAN_BIOSYNTHESIS_HEPARAN_SULFATE | 26 | 0.57 | 1.78 | 0.01 | 0.01 |
| KEGG_LYSOSOME | 116 | 0.51 | 1.77 | 0.01 | 0.02 |
| KEGG_VASCULAR_SMOOTH_MUSCLE_CONTRACTION | 103 | 0.53 | 1.77 | 0.00 | 0.02 |
| KEGG_BASAL_CELL_CARCINOMA | 52 | 0.58 | 1.75 | 0.00 | 0.02 |
| KEGG_SYSTEMIC_LUPUS_ERYTHEMATOSUS | 50 | 0.79 | 1.72 | 0.00 | 0.03 |
| KEGG_PROSTATE_CANCER | 89 | 0.48 | 1.71 | 0.00 | 0.02 |
| KEGG_EPITHELIAL_CELL_SIGNALING_IN_HELICOBACTER_PYLORI_INFECTION | 67 | 0.48 | 1.71 | 0.01 | 0.03 |
| KEGG_GAP_JUNCTION | 84 | 0.52 | 1.70 | 0.00 | 0.03 |
| KEGG_FC_GAMMA_R_MEDIATED_PHAGOCYTOSIS | 90 | 0.51 | 1.67 | 0.00 | 0.04 |
| KEGG_RENAL_CELL_CARCINOMA | 66 | 0.50 | 1.67 | 0.00 | 0.03 |
| KEGG_ASTHMA | 26 | 0.77 | 1.66 | 0.00 | 0.04 |
| KEGG_TGF_BETA_SIGNALING_PATHWAY | 81 | 0.53 | 1.66 | 0.00 | 0.04 |
| KEGG_BLADDER_CANCER | 41 | 0.61 | 1.66 | 0.00 | 0.03 |
| KEGG_PRION_DISEASES | 34 | 0.68 | 1.66 | 0.01 | 0.04 |
| KEGG_JAK_STAT_SIGNALING_PATHWAY | 152 | 0.47 | 1.66 | 0.01 | 0.03 |
| KEGG_PATHOGENIC_ESCHERICHIA_COLI_INFECTION | 51 | 0.55 | 1.65 | 0.03 | 0.04 |
| KEGG_GRAFT_VERSUS_HOST_DISEASE | 35 | 0.77 | 1.64 | 0.01 | 0.04 |
| KEGG_TIGHT_JUNCTION | 125 | 0.45 | 1.64 | 0.00 | 0.04 |
| KEGG_ADHERENS_JUNCTION | 66 | 0.52 | 1.64 | 0.01 | 0.04 |
| KEGG_CYTOKINE_CYTOKINE_RECEPTOR_INTERACTION | 250 | 0.56 | 1.63 | 0.00 | 0.04 |
| KEGG_AUTOIMMUNE_THYROID_DISEASE | 46 | 0.65 | 1.63 | 0.01 | 0.04 |
| KEGG_NATURAL_KILLER_CELL_MEDIATED_CYTOTOXICITY | 128 | 0.53 | 1.62 | 0.01 | 0.04 |
| KEGG_TYPE_I_DIABETES_MELLITUS | 39 | 0.70 | 1.62 | 0.01 | 0.04 |
| KEGG_PANCREATIC_CANCER | 69 | 0.47 | 1.61 | 0.01 | 0.04 |
| KEGG_ACUTE_MYELOID_LEUKEMIA | 56 | 0.47 | 1.60 | 0.01 | 0.05 |

**Supplementary Table 10.** GSEA results of KEGG pathway enrichment in CRC patients with high FN1 expression in the GSE17536 dataset

| **NAME** | **SIZE** | **ES** | **NES** | **NOM p-val** | **FDR q-val** |
| --- | --- | --- | --- | --- | --- |
| KEGG_LYSOSOME | 116 | 0.62 | 1.87 | 0.00 | 0.04 |
| KEGG_FOCAL_ADHESION | 193 | 0.66 | 1.90 | 0.00 | 0.05 |
| KEGG_PATHWAYS_IN_CANCER | 318 | 0.50 | 1.71 | 0.00 | 0.06 |
| KEGG_PROSTATE_CANCER | 89 | 0.54 | 1.72 | 0.01 | 0.06 |
| KEGG_REGULATION_OF_ACTIN_CYTOSKELETON | 207 | 0.48 | 1.71 | 0.00 | 0.06 |
| KEGG_PANCREATIC_CANCER | 69 | 0.55 | 1.69 | 0.00 | 0.06 |
| KEGG_LEUKOCYTE_TRANSENDOTHELIAL_MIGRATION | 111 | 0.60 | 1.69 | 0.00 | 0.07 |
| KEGG_UBIQUITIN_MEDIATED_PROTEOLYSIS | 125 | 0.52 | 1.72 | 0.00 | 0.07 |
| KEGG_CHRONIC_MYELOID_LEUKEMIA | 72 | 0.53 | 1.73 | 0.00 | 0.07 |
| KEGG_FC_GAMMA_R_MEDIATED_PHAGOCYTOSIS | 90 | 0.54 | 1.67 | 0.01 | 0.08 |
| KEGG_PATHOGENIC_ESCHERICHIA_COLI_INFECTION | 51 | 0.66 | 1.73 | 0.00 | 0.08 |
| KEGG_ACUTE_MYELOID_LEUKEMIA | 56 | 0.56 | 1.75 | 0.00 | 0.08 |
| KEGG_TGF_BETA_SIGNALING_PATHWAY | 81 | 0.59 | 1.65 | 0.01 | 0.08 |
| KEGG_ADHERENS_JUNCTION | 66 | 0.63 | 1.77 | 0.00 | 0.08 |
| KEGG_ECM_RECEPTOR_INTERACTION | 81 | 0.72 | 1.73 | 0.01 | 0.09 |
| KEGG_MTOR_SIGNALING_PATHWAY | 50 | 0.55 | 1.79 | 0.00 | 0.09 |
| KEGG_COLORECTAL_CANCER | 62 | 0.54 | 1.63 | 0.00 | 0.09 |
| KEGG_NEUROTROPHIN_SIGNALING_PATHWAY | 122 | 0.44 | 1.64 | 0.00 | 0.09 |
| KEGG_RENAL_CELL_CARCINOMA | 66 | 0.53 | 1.63 | 0.01 | 0.09 |
| KEGG_SMALL_CELL_LUNG_CANCER | 84 | 0.57 | 1.75 | 0.00 | 0.10 |
| KEGG_AXON_GUIDANCE | 128 | 0.45 | 1.58 | 0.01 | 0.12 |
| KEGG_GLYCOSAMINOGLYCAN_BIOSYNTHESIS_CHONDROITIN_SULFATE | 22 | 0.65 | 1.58 | 0.05 | 0.12 |
| KEGG_ARRHYTHMOGENIC_RIGHT_VENTRICULAR_CARDIOMYOPATHY_ARVC | 72 | 0.51 | 1.57 | 0.03 | 0.12 |
| KEGG_ENDOMETRIAL_CANCER | 52 | 0.57 | 1.58 | 0.01 | 0.13 |
| KEGG_PROTEIN_EXPORT | 22 | 0.61 | 1.57 | 0.06 | 0.13 |
| KEGG_VASOPRESSIN_REGULATED_WATER_REABSORPTION | 44 | 0.47 | 1.56 | 0.02 | 0.13 |
| KEGG_GLIOMA | 63 | 0.50 | 1.59 | 0.02 | 0.13 |
| KEGG_TIGHT_JUNCTION | 125 | 0.46 | 1.54 | 0.01 | 0.15 |
| KEGG_GAP_JUNCTION | 84 | 0.50 | 1.51 | 0.02 | 0.15 |
| KEGG_ENDOCYTOSIS | 170 | 0.41 | 1.52 | 0.01 | 0.15 |
| KEGG_MELANOMA | 71 | 0.50 | 1.51 | 0.03 | 0.15 |
| KEGG_CIRCADIAN_RHYTHM_MAMMAL | 10 | 0.70 | 1.52 | 0.04 | 0.16 |
| KEGG_BLADDER_CANCER | 41 | 0.56 | 1.51 | 0.02 | 0.16 |
| KEGG_HYPERTROPHIC_CARDIOMYOPATHY_HCM | 81 | 0.47 | 1.52 | 0.04 | 0.16 |
| KEGG_GLYCOSPHINGOLIPID_BIOSYNTHESIS_GLOBO_SERIES | 14 | 0.62 | 1.52 | 0.05 | 0.16 |
| KEGG_TOLL_LIKE_RECEPTOR_SIGNALING_PATHWAY | 97 | 0.56 | 1.49 | 0.07 | 0.17 |
| KEGG_B_CELL_RECEPTOR_SIGNALING_PATHWAY | 75 | 0.51 | 1.48 | 0.04 | 0.17 |
| KEGG_NON_SMALL_CELL_LUNG_CANCER | 54 | 0.46 | 1.46 | 0.03 | 0.18 |
| KEGG_ERBB_SIGNALING_PATHWAY | 86 | 0.42 | 1.43 | 0.04 | 0.18 |
| KEGG_THYROID_CANCER | 29 | 0.58 | 1.47 | 0.06 | 0.18 |
| KEGG_VASCULAR_SMOOTH_MUSCLE_CONTRACTION | 103 | 0.47 | 1.47 | 0.06 | 0.18 |
| KEGG_PRION_DISEASES | 34 | 0.58 | 1.43 | 0.07 | 0.18 |
| KEGG_GLYCOSAMINOGLYCAN_DEGRADATION | 20 | 0.53 | 1.43 | 0.07 | 0.18 |
| KEGG_VIRAL_MYOCARDITIS | 65 | 0.57 | 1.44 | 0.08 | 0.19 |
| KEGG_CELL_ADHESION_MOLECULES_CAMS | 125 | 0.52 | 1.45 | 0.06 | 0.19 |
| KEGG_WNT_SIGNALING_PATHWAY | 146 | 0.45 | 1.44 | 0.02 | 0.19 |
| KEGG_SPHINGOLIPID_METABOLISM | 31 | 0.60 | 1.42 | 0.06 | 0.19 |
| KEGG_DILATED_CARDIOMYOPATHY | 88 | 0.46 | 1.44 | 0.08 | 0.19 |
| KEGG_INSULIN_SIGNALING_PATHWAY | 133 | 0.40 | 1.41 | 0.06 | 0.19 |
| KEGG_LEISHMANIA_INFECTION | 67 | 0.60 | 1.44 | 0.09 | 0.19 |
| KEGG_EPITHELIAL_CELL_SIGNALING_IN_HELICOBACTER_PYLORI_INFECTION | 67 | 0.44 | 1.44 | 0.04 | 0.20 |
| KEGG_MAPK_SIGNALING_PATHWAY | 254 | 0.38 | 1.40 | 0.03 | 0.21 |
| KEGG_APOPTOSIS | 87 | 0.44 | 1.38 | 0.07 | 0.22 |
| KEGG_NOD_LIKE_RECEPTOR_SIGNALING_PATHWAY | 60 | 0.53 | 1.38 | 0.11 | 0.22 |
| KEGG_BASAL_TRANSCRIPTION_FACTORS | 32 | 0.46 | 1.37 | 0.10 | 0.23 |
| KEGG_VEGF_SIGNALING_PATHWAY | 71 | 0.44 | 1.36 | 0.07 | 0.24 |

**Supplementary Table 11.** GSEA results of KEGG pathway enrichment in CRC patients with high FN1 expression in the GSE39582 dataset

| **NAME** | **SIZE** | **ES** | **NES** | **NOM p-val** | **FDR q-val** |
| --- | --- | --- | --- | --- | --- |
| KEGG_FOCAL_ADHESION | 193 | 0.66 | 1.95 | 0.00 | 0.01 |
| KEGG_ECM_RECEPTOR_INTERACTION | 81 | 0.80 | 1.99 | 0.00 | 0.01 |
| KEGG_HYPERTROPHIC_CARDIOMYOPATHY_HCM | 81 | 0.59 | 1.87 | 0.00 | 0.02 |
| KEGG_GLYCOSAMINOGLYCAN_BIOSYNTHESIS_CHONDROITIN_SULFATE | 22 | 0.77 | 1.79 | 0.00 | 0.05 |
| KEGG_DILATED_CARDIOMYOPATHY | 88 | 0.55 | 1.75 | 0.01 | 0.07 |
| KEGG_TGF_BETA_SIGNALING_PATHWAY | 81 | 0.55 | 1.73 | 0.00 | 0.07 |
| KEGG_ARRHYTHMOGENIC_RIGHT_VENTRICULAR_CARDIOMYOPATHY_ARVC | 72 | 0.54 | 1.71 | 0.01 | 0.07 |
| KEGG_AXON_GUIDANCE | 128 | 0.46 | 1.70 | 0.01 | 0.08 |
| KEGG_CELL_ADHESION_MOLECULES_CAMS | 125 | 0.57 | 1.57 | 0.02 | 0.18 |
| KEGG_VASCULAR_SMOOTH_MUSCLE_CONTRACTION | 103 | 0.46 | 1.53 | 0.03 | 0.18 |
| KEGG_REGULATION_OF_ACTIN_CYTOSKELETON | 207 | 0.41 | 1.52 | 0.01 | 0.19 |
| KEGG_MELANOMA | 71 | 0.49 | 1.53 | 0.01 | 0.19 |
| KEGG_SMALL_CELL_LUNG_CANCER | 84 | 0.47 | 1.58 | 0.01 | 0.19 |
| KEGG_VIRAL_MYOCARDITIS | 65 | 0.57 | 1.51 | 0.04 | 0.19 |
| KEGG_WNT_SIGNALING_PATHWAY | 146 | 0.45 | 1.59 | 0.00 | 0.19 |
| KEGG_PATHWAYS_IN_CANCER | 318 | 0.41 | 1.54 | 0.00 | 0.20 |
| KEGG_MTOR_SIGNALING_PATHWAY | 50 | 0.43 | 1.54 | 0.01 | 0.21 |
| KEGG_GLIOMA | 63 | 0.43 | 1.47 | 0.02 | 0.23 |

**Supplementary Table 12.** GSEA results of KEGG pathway enrichment in CRC patients with high COL1A1 expression in the GSE17536 dataset

| **NAME** | **SIZE** | **ES** | **NES** | **NOM p-val** | **FDR**  **q-val** |
| --- | --- | --- | --- | --- | --- |
| KEGG_REGULATION_OF_ACTIN_CYTOSKELETON | 207 | 0.53 | 1.87 | 0.00 | 0.02 |
| KEGG_LYSOSOME | 116 | 0.61 | 1.89 | 0.00 | 0.02 |
| KEGG_FOCAL_ADHESION | 193 | 0.71 | 1.97 | 0.00 | 0.02 |
| KEGG_ECM_RECEPTOR_INTERACTION | 81 | 0.81 | 1.91 | 0.00 | 0.02 |
| KEGG_HYPERTROPHIC_CARDIOMYOPATHY_HCM | 81 | 0.55 | 1.75 | 0.00 | 0.03 |
| KEGG_FC_GAMMA_R_MEDIATED_PHAGOCYTOSIS | 90 | 0.58 | 1.74 | 0.00 | 0.03 |
| KEGG_DILATED_CARDIOMYOPATHY | 88 | 0.58 | 1.79 | 0.00 | 0.03 |
| KEGG_CELL_ADHESION_MOLECULES_CAMS | 125 | 0.63 | 1.75 | 0.00 | 0.03 |
| KEGG_MTOR_SIGNALING_PATHWAY | 50 | 0.56 | 1.80 | 0.00 | 0.03 |
| KEGG_VASCULAR_SMOOTH_MUSCLE_CONTRACTION | 103 | 0.56 | 1.77 | 0.00 | 0.03 |
| KEGG_SMALL_CELL_LUNG_CANCER | 84 | 0.57 | 1.74 | 0.00 | 0.03 |
| KEGG_ARRHYTHMOGENIC_RIGHT_VENTRICULAR_CARDIOMYOPATHY_ARVC | 72 | 0.57 | 1.76 | 0.00 | 0.03 |
| KEGG_PROSTATE_CANCER | 89 | 0.54 | 1.72 | 0.00 | 0.03 |
| KEGG_ACUTE_MYELOID_LEUKEMIA | 56 | 0.55 | 1.72 | 0.00 | 0.04 |
| KEGG_AXON_GUIDANCE | 128 | 0.53 | 1.77 | 0.00 | 0.04 |
| KEGG_PATHWAYS_IN_CANCER | 318 | 0.50 | 1.71 | 0.00 | 0.04 |
| KEGG_GLIOMA | 63 | 0.58 | 1.80 | 0.00 | 0.04 |
| KEGG_LEUKOCYTE_TRANSENDOTHELIAL_MIGRATION | 111 | 0.61 | 1.69 | 0.00 | 0.04 |
| KEGG_GAP_JUNCTION | 84 | 0.56 | 1.69 | 0.00 | 0.04 |
| KEGG_EPITHELIAL_CELL_SIGNALING_IN_HELICOBACTER_PYLORI_INFECTION | 67 | 0.52 | 1.68 | 0.00 | 0.04 |
| KEGG_GLYCOSAMINOGLYCAN_BIOSYNTHESIS_CHONDROITIN_SULFATE | 22 | 0.71 | 1.69 | 0.01 | 0.04 |
| KEGG_TOLL_LIKE_RECEPTOR_SIGNALING_PATHWAY | 97 | 0.62 | 1.64 | 0.01 | 0.06 |

**Supplementary Table 13.** GSEA results of KEGG pathway enrichment in CRC patients with high COL1A1 expression in the GSE39582 dataset

| **NAME** | **SIZE** | **ES** | **NES** | **NOM p-val** | **FDR q-val** |
| --- | --- | --- | --- | --- | --- |
| KEGG_FOCAL_ADHESION | 193 | 0.75 | 2.20 | 0.00 | 0.00 |
| KEGG_ECM_RECEPTOR_INTERACTION | 81 | 0.85 | 2.09 | 0.00 | 0.00 |
| KEGG_HYPERTROPHIC_CARDIOMYOPATHY_HCM | 81 | 0.64 | 2.08 | 0.00 | 0.00 |
| KEGG_DILATED_CARDIOMYOPATHY | 88 | 0.64 | 2.03 | 0.00 | 0.00 |
| KEGG_GLYCOSAMINOGLYCAN_BIOSYNTHESIS_CHONDROITIN_SULFATE | 22 | 0.83 | 2.01 | 0.00 | 0.00 |
| KEGG_GLYCOSAMINOGLYCAN_BIOSYNTHESIS_HEPARAN_SULFATE | 26 | 0.64 | 1.99 | 0.00 | 0.00 |
| KEGG_MTOR_SIGNALING_PATHWAY | 50 | 0.56 | 1.98 | 0.00 | 0.00 |
| KEGG_ARRHYTHMOGENIC_RIGHT_VENTRICULAR_CARDIOMYOPATHY_ARVC | 72 | 0.62 | 1.97 | 0.00 | 0.00 |
| KEGG_PATHWAYS_IN_CANCER | 318 | 0.53 | 1.96 | 0.00 | 0.00 |
| KEGG_SMALL_CELL_LUNG_CANCER | 84 | 0.57 | 1.95 | 0.00 | 0.00 |
| KEGG_AXON_GUIDANCE | 128 | 0.54 | 1.94 | 0.00 | 0.00 |
| KEGG_REGULATION_OF_ACTIN_CYTOSKELETON | 207 | 0.52 | 1.90 | 0.00 | 0.00 |
| KEGG_CELL_ADHESION_MOLECULES_CAMS | 125 | 0.70 | 1.88 | 0.00 | 0.01 |
| KEGG_VASCULAR_SMOOTH_MUSCLE_CONTRACTION | 103 | 0.56 | 1.86 | 0.00 | 0.01 |
| KEGG_BASAL_CELL_CARCINOMA | 52 | 0.61 | 1.85 | 0.00 | 0.01 |
| KEGG_WNT_SIGNALING_PATHWAY | 146 | 0.52 | 1.83 | 0.00 | 0.01 |
| KEGG_MELANOMA | 71 | 0.57 | 1.81 | 0.00 | 0.01 |
| KEGG_VIRAL_MYOCARDITIS | 65 | 0.68 | 1.77 | 0.00 | 0.02 |
| KEGG_GLIOMA | 63 | 0.52 | 1.75 | 0.00 | 0.02 |
| KEGG_ADHERENS_JUNCTION | 66 | 0.56 | 1.74 | 0.00 | 0.02 |
| KEGG_LEUKOCYTE_TRANSENDOTHELIAL_MIGRATION | 111 | 0.62 | 1.72 | 0.00 | 0.03 |
| KEGG_GAP_JUNCTION | 84 | 0.53 | 1.71 | 0.00 | 0.03 |
| KEGG_PROSTATE_CANCER | 89 | 0.49 | 1.71 | 0.00 | 0.03 |
| KEGG_NOTCH_SIGNALING_PATHWAY | 46 | 0.42 | 1.70 | 0.01 | 0.03 |
| KEGG_LEISHMANIA_INFECTION | 67 | 0.71 | 1.70 | 0.01 | 0.03 |
| KEGG_TOLL_LIKE_RECEPTOR_SIGNALING_PATHWAY | 97 | 0.62 | 1.68 | 0.00 | 0.04 |
| KEGG_GLYCOSAMINOGLYCAN_DEGRADATION | 20 | 0.64 | 1.66 | 0.01 | 0.04 |
| KEGG_EPITHELIAL_CELL_SIGNALING_IN_HELICOBACTER_PYLORI_INFECTION | 67 | 0.47 | 1.65 | 0.01 | 0.04 |
| KEGG_MELANOGENESIS | 96 | 0.47 | 1.64 | 0.01 | 0.05 |
| KEGG_SYSTEMIC_LUPUS_ERYTHEMATOSUS | 50 | 0.75 | 1.64 | 0.01 | 0.04 |
| KEGG_ASTHMA | 26 | 0.75 | 1.64 | 0.01 | 0.04 |
| KEGG_CYTOKINE_CYTOKINE_RECEPTOR_INTERACTION | 250 | 0.57 | 1.64 | 0.01 | 0.04 |
| KEGG_JAK_STAT_SIGNALING_PATHWAY | 152 | 0.47 | 1.63 | 0.01 | 0.04 |
| KEGG_BLADDER_CANCER | 41 | 0.59 | 1.63 | 0.01 | 0.04 |
| KEGG_TIGHT_JUNCTION | 125 | 0.46 | 1.62 | 0.00 | 0.05 |

**Supplementary Table 14.** GSEA results of KEGG pathway enrichment in CRC patients with high COL5A1 expression in the GSE17536 dataset

| **NAME** | **SIZE** | **ES** | **NES** | **NOM p-val** | **FDR q-val** |
| --- | --- | --- | --- | --- | --- |
| KEGG_FOCAL_ADHESION | 193 | 0.70 | 1.99 | 0.00 | 0.02 |
| KEGG_REGULATION_OF_ACTIN_CYTOSKELETON | 207 | 0.54 | 1.93 | 0.00 | 0.02 |
| KEGG_DILATED_CARDIOMYOPATHY | 88 | 0.61 | 1.92 | 0.00 | 0.01 |
| KEGG_ECM_RECEPTOR_INTERACTION | 81 | 0.80 | 1.92 | 0.00 | 0.01 |
| KEGG_HYPERTROPHIC_CARDIOMYOPATHY_HCM | 81 | 0.59 | 1.91 | 0.00 | 0.01 |
| KEGG_MTOR_SIGNALING_PATHWAY | 50 | 0.57 | 1.89 | 0.00 | 0.01 |
| KEGG_CELL_ADHESION_MOLECULES_CAMS | 125 | 0.66 | 1.88 | 0.00 | 0.01 |
| KEGG_VASCULAR_SMOOTH_MUSCLE_CONTRACTION | 103 | 0.59 | 1.88 | 0.00 | 0.01 |
| KEGG_ARRHYTHMOGENIC_RIGHT_VENTRICULAR_CARDIOMYOPATHY_ARVC | 72 | 0.59 | 1.83 | 0.00 | 0.02 |
| KEGG_GLIOMA | 63 | 0.55 | 1.78 | 0.00 | 0.03 |
| KEGG_GAP_JUNCTION | 84 | 0.59 | 1.77 | 0.00 | 0.03 |
| KEGG_VIRAL_MYOCARDITIS | 65 | 0.69 | 1.77 | 0.00 | 0.02 |
| KEGG_GLYCOSAMINOGLYCAN_BIOSYNTHESIS_CHONDROITIN_SULFATE | 22 | 0.72 | 1.76 | 0.01 | 0.02 |
| KEGG_LYSOSOME | 116 | 0.57 | 1.76 | 0.00 | 0.02 |
| KEGG_LEUKOCYTE_TRANSENDOTHELIAL_MIGRATION | 111 | 0.61 | 1.76 | 0.00 | 0.02 |
| KEGG_PATHWAYS_IN_CANCER | 318 | 0.51 | 1.76 | 0.00 | 0.02 |
| KEGG_PROSTATE_CANCER | 89 | 0.54 | 1.74 | 0.00 | 0.03 |
| KEGG_FC_GAMMA_R_MEDIATED_PHAGOCYTOSIS | 90 | 0.57 | 1.73 | 0.00 | 0.03 |
| KEGG_AXON_GUIDANCE | 128 | 0.50 | 1.72 | 0.00 | 0.03 |
| KEGG_MELANOMA | 71 | 0.57 | 1.71 | 0.00 | 0.03 |
| KEGG_SMALL_CELL_LUNG_CANCER | 84 | 0.57 | 1.70 | 0.00 | 0.03 |
| KEGG_RENAL_CELL_CARCINOMA | 66 | 0.55 | 1.69 | 0.00 | 0.04 |
| KEGG_ACUTE_MYELOID_LEUKEMIA | 56 | 0.53 | 1.68 | 0.00 | 0.04 |
| KEGG_MAPK_SIGNALING_PATHWAY | 254 | 0.45 | 1.67 | 0.00 | 0.04 |
| KEGG_TOLL_LIKE_RECEPTOR_SIGNALING_PATHWAY | 97 | 0.62 | 1.66 | 0.01 | 0.04 |
| KEGG_EPITHELIAL_CELL_SIGNALING_IN_HELICOBACTER_PYLORI_INFECTION | 67 | 0.50 | 1.64 | 0.01 | 0.05 |

**Supplementary Table 15.** GSEA results of KEGG pathway enrichment in CRC patients with high COL5A1 expression in the GSE39582 dataset

| **NAME** | **SIZE** | **ES** | **NES** | **NOM p-val** | **FDR**  **q-val** |
| --- | --- | --- | --- | --- | --- |
| KEGG_FOCAL_ADHESION | 193 | 0.74 | 2.22 | 0.00 | 0.00 |
| KEGG_HYPERTROPHIC_CARDIOMYOPATHY_HCM | 81 | 0.64 | 2.04 | 0.00 | 0.00 |
| KEGG_ECM_RECEPTOR_INTERACTION | 81 | 0.84 | 2.05 | 0.00 | 0.00 |
| KEGG_PATHWAYS_IN_CANCER | 318 | 0.54 | 2.02 | 0.00 | 0.00 |
| KEGG_AXON_GUIDANCE | 128 | 0.56 | 2.03 | 0.00 | 0.00 |
| KEGG_MELANOMA | 71 | 0.61 | 1.98 | 0.00 | 0.00 |
| KEGG_MTOR_SIGNALING_PATHWAY | 50 | 0.59 | 2.10 | 0.00 | 0.00 |
| KEGG_REGULATION_OF_ACTIN_CYTOSKELETON | 207 | 0.53 | 1.99 | 0.00 | 0.00 |
| KEGG_CELL_ADHESION_MOLECULES_CAMS | 125 | 0.72 | 1.97 | 0.00 | 0.00 |
| KEGG_GLYCOSAMINOGLYCAN_BIOSYNTHESIS_CHONDROITIN_SULFATE | 22 | 0.83 | 1.99 | 0.00 | 0.00 |
| KEGG_SMALL_CELL_LUNG_CANCER | 84 | 0.58 | 1.96 | 0.00 | 0.00 |
| KEGG_ARRHYTHMOGENIC_RIGHT_VENTRICULAR_CARDIOMYOPATHY_ARVC | 72 | 0.61 | 1.95 | 0.00 | 0.00 |
| KEGG_DILATED_CARDIOMYOPATHY | 88 | 0.63 | 1.99 | 0.00 | 0.00 |
| KEGG_VASCULAR_SMOOTH_MUSCLE_CONTRACTION | 103 | 0.56 | 1.91 | 0.00 | 0.00 |
| KEGG_WNT_SIGNALING_PATHWAY | 146 | 0.53 | 1.89 | 0.00 | 0.00 |
| KEGG_LEUKOCYTE_TRANSENDOTHELIAL_MIGRATION | 111 | 0.65 | 1.87 | 0.00 | 0.00 |
| KEGG_LYSOSOME | 116 | 0.53 | 1.84 | 0.01 | 0.01 |
| KEGG_NOTCH_SIGNALING_PATHWAY | 46 | 0.44 | 1.82 | 0.00 | 0.01 |
| KEGG_PROSTATE_CANCER | 89 | 0.51 | 1.81 | 0.00 | 0.01 |
| KEGG_VIRAL_MYOCARDITIS | 65 | 0.70 | 1.82 | 0.00 | 0.01 |
| KEGG_GLYCOSAMINOGLYCAN_BIOSYNTHESIS_HEPARAN_SULFATE | 26 | 0.57 | 1.81 | 0.00 | 0.01 |
| KEGG_BASAL_CELL_CARCINOMA | 52 | 0.61 | 1.82 | 0.00 | 0.01 |
| KEGG_LEISHMANIA_INFECTION | 67 | 0.74 | 1.82 | 0.00 | 0.01 |
| KEGG_GLIOMA | 63 | 0.53 | 1.83 | 0.00 | 0.01 |
| KEGG_TOLL_LIKE_RECEPTOR_SIGNALING_PATHWAY | 97 | 0.64 | 1.82 | 0.00 | 0.01 |
| KEGG_ANTIGEN_PROCESSING_AND_PRESENTATION | 74 | 0.60 | 1.79 | 0.01 | 0.01 |
| KEGG_GAP_JUNCTION | 84 | 0.54 | 1.78 | 0.00 | 0.01 |
| KEGG_BLADDER_CANCER | 41 | 0.63 | 1.77 | 0.00 | 0.01 |
| KEGG_RENAL_CELL_CARCINOMA | 66 | 0.53 | 1.75 | 0.00 | 0.02 |
| KEGG_SYSTEMIC_LUPUS_ERYTHEMATOSUS | 50 | 0.78 | 1.75 | 0.00 | 0.02 |
| KEGG_JAK_STAT_SIGNALING_PATHWAY | 152 | 0.49 | 1.75 | 0.00 | 0.02 |
| KEGG_MELANOGENESIS | 96 | 0.49 | 1.74 | 0.00 | 0.02 |
| KEGG_TIGHT_JUNCTION | 125 | 0.48 | 1.73 | 0.00 | 0.02 |
| KEGG_GLYCOSAMINOGLYCAN_DEGRADATION | 20 | 0.65 | 1.72 | 0.01 | 0.02 |
| KEGG_EPITHELIAL_CELL_SIGNALING_IN_HELICOBACTER_PYLORI_INFECTION | 67 | 0.49 | 1.71 | 0.00 | 0.02 |
| KEGG_PATHOGENIC_ESCHERICHIA_COLI_INFECTION | 51 | 0.58 | 1.71 | 0.01 | 0.02 |
| KEGG_ADHERENS_JUNCTION | 66 | 0.55 | 1.71 | 0.00 | 0.02 |
| KEGG_CYTOKINE_CYTOKINE_RECEPTOR_INTERACTION | 250 | 0.59 | 1.70 | 0.00 | 0.02 |
| KEGG_MAPK_SIGNALING_PATHWAY | 254 | 0.43 | 1.70 | 0.00 | 0.02 |
| KEGG_ASTHMA | 26 | 0.77 | 1.68 | 0.00 | 0.03 |
| KEGG_CHEMOKINE_SIGNALING_PATHWAY | 179 | 0.56 | 1.66 | 0.01 | 0.03 |
| KEGG_GRAFT_VERSUS_HOST_DISEASE | 35 | 0.76 | 1.66 | 0.02 | 0.03 |
| KEGG_TGF_BETA_SIGNALING_PATHWAY | 81 | 0.51 | 1.66 | 0.01 | 0.03 |
| KEGG_AUTOIMMUNE_THYROID_DISEASE | 46 | 0.62 | 1.65 | 0.03 | 0.03 |
| KEGG_NATURAL_KILLER_CELL_MEDIATED_CYTOTOXICITY | 128 | 0.53 | 1.65 | 0.03 | 0.03 |
| KEGG_PANCREATIC_CANCER | 69 | 0.48 | 1.64 | 0.01 | 0.03 |
| KEGG_FC_GAMMA_R_MEDIATED_PHAGOCYTOSIS | 90 | 0.51 | 1.64 | 0.01 | 0.03 |
| KEGG_PRION_DISEASES | 34 | 0.67 | 1.63 | 0.01 | 0.03 |
| KEGG_TYPE_I_DIABETES_MELLITUS | 39 | 0.68 | 1.63 | 0.02 | 0.03 |
| KEGG_ACUTE_MYELOID_LEUKEMIA | 56 | 0.48 | 1.59 | 0.02 | 0.05 |
