# Supplementary material for: Transformative insights from transcriptome analysis of colorectal cancer patient tissues: identification of four key prognostic genes
Source: PeerJ. 2025 Aug 20;13:e19852. doi: 10.7717/peerj.19852 (PMC12374693; doi:10.7717/peerj.19852)
Supplement: Supplemental Information 2 [file peerj-13-19852-s002.pdf]

Supplementary Figures

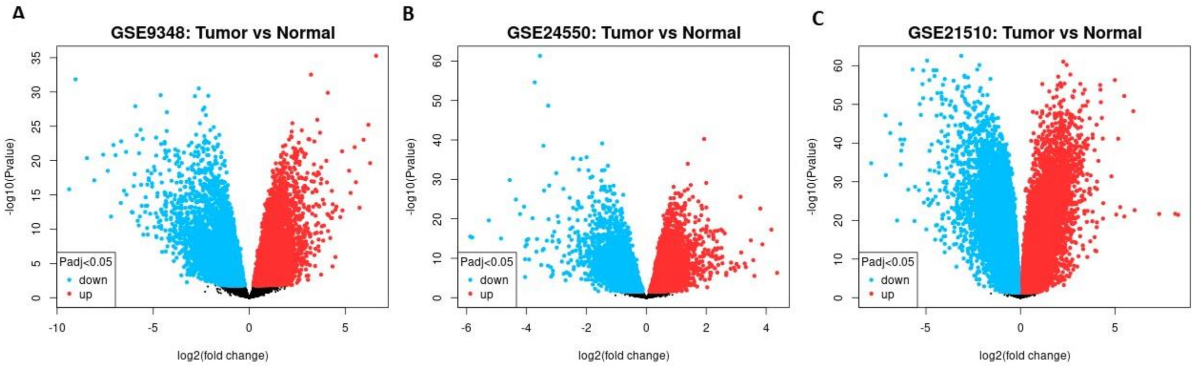

Supplementary Figure 1: Differential expressed genes between colorectal cancer and normal samples in three independent datasets. DEGs were displayed by volcano plots in (A) GSE9348, (B) GSE24550, and (C) GSE21510. Each dot represents a gene, with red indicating up-regulated genes, blue indicating down-regulated genes, and black indicating genes with no significance.

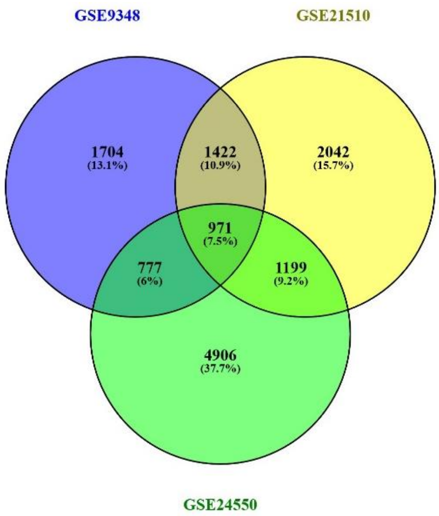

Supplementary Figure 2: Common differential expressed genes in three datasets from the integrated microarray dataset analysis.

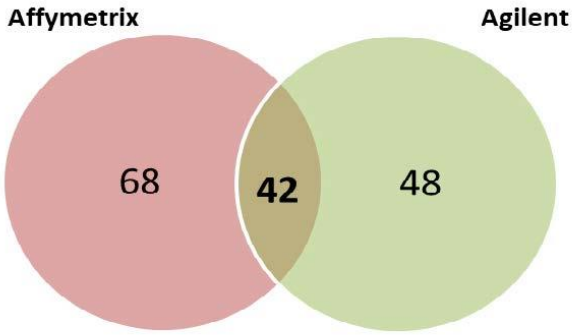

Supplementary Figure 3: Common differential expressed miRNAs in the datasets from the Affymetrix and Agilent microarray platforms.
