# Supplementary material for: Transformative insights from transcriptome analysis of colorectal cancer patient tissues: identification of four key prognostic genes
Source: PeerJ. 2025 Aug 20;13:e19852. doi: 10.7717/peerj.19852 (PMC12374693; doi:10.7717/peerj.19852)
Supplement: Supplemental Information 3 [file peerj-13-19852-s003.docx]

| ITEM TO CHECK | IMPORTANCE | DETAILS |
| --- | --- | --- |
| **EXPERIMENTAL DESIGN** |  |  |
| Definition of experimental and control groups | E | The experimental group consisted of two cohorts. The discovery cohort included 49 matched tumour and normal FFPE sporadic colorectal cancer tissue samples, used for transcriptome profiling, while the validation cohort comprised 64 tumour and matched normal CRC samples for validating and testing the reproducibility of the expression results. （Line77-84） |
| Number within each group | E | The discovery cohort included 49 matched tumour and normal FFPE sporadic colorectal cancer tissue samples, and the validation cohort comprised 64 matched tumour and normal CRC samples (Lines 77-84). |
| Assay carried out by core lab or investigator's lab? | D | The assays were carried out in the investigator's lab. |
| Acknowledgement of authors' contributions | D | Acknowledgement of authors' contributions is provided in the document. |
| **SAMPLE** |  |  |
| Description | E | Formalin-fixed paraffin-embedded (FFPE) matched tumour and normal tissue samples from sporadic colorectal cancer cases (Line 66,67, 77-84） |
| Volume/mass of sample processed | D | Four 8-μm sections per block were cut, placed on slides and used for RNA isolation（Line 88） |
| Microdissection or macrodissection | E | The marked tumor regions were macrodissected from serial sections. （Line 92). |
| Processing procedure | E | The FFPE tissue samples were fixed in formalin immediately after collection, embedded in paraffin blocks. (Lines 85-86). |
| If frozen - how and how quickly? | E |  |
| If fixed - with what, how quickly? | E | The FFPE tissue samples were fixed in 10% neutral-buffered formalin immediately after collection, embedded in paraffin blocks (Lines 85-86). |
| Sample storage conditions and duration (especially for FFPE) | E | FFPE samples were were stored at -20°C until used for transcriptome profiling analysis. (Lines 89). |
| **NUCLEIC ACID EXTRACTION** |  |  |
| Procedure and/or instrumentation | E | RNA extraction was performed using RNeasy FFPE kit. (Line92-94） |
| Name of kit and details of any modifications | E | RNA extraction was performed using RNeasy FFPE kit (Qiagen, Hilden, Germany), following a modified deparaffinization step as previously described (Belder et al. 2016)（Line92-94） |
| Source of additional reagents used | D | Additional reagents were sourced from Invitrogen and Sigma. |
| Details of DNase or RNAse treatment | E | Genomic DNA was removed using the gDNA Eliminator spin column included in the kit (Line 95). |
| Contamination assessment (DNA or RNA) | D | Contamination was assessed using no-RT controls in qPCR. |
| Nucleic acid quantification | E | Quantification was performed using a NanoDrop spectrophotometer. |
| Instrument and method | E | RNA quantity and purity were measured using a NanoDrop spectrophotometer. |
| Purity (A260/A280) | D | RNA purity was assessed by measuring the A260/A280 ratio, aiming for values between 1.8 and 2.0. |
| Yield | D | RNA yield from macrodissected sections was typically in the range of 24.68 to 445.65 ng/µL. |
| RNA integrity method/instrument | E | RNA integrity was assessed using an Agilent 2100 Bioanalyzer. |
| RIN/RQI or Cq of 3' and 5' transcripts | E | RNA Integrity Number (RIN) values were araound 2.5. （Line120-127） |
| Electrophoresis traces | D |  |
| Inhibition testing (Cq dilutions, spike or other) | E | No specific inhibition testing (e.g., Cq dilutions, spike) was performed. However, the SYBR Green I Master Mix was used according to the manufacturer’s instructions, and all reactions were carried out in triplicate to ensure the accuracy and reproducibility of the results. No significant inhibition was observed in the amplification process |
| **REVERSE TRANSCRIPTION** |  |  |
| Complete reaction conditions | E | Reverse transcription was performed using the Transcriptor Kit (Roche, Basel, Switzerland), with specific conditions detailed below. (Line 174-175) |
| Amount of RNA and reaction volume | E | 0.5 µg of RNA was used in a 20 µL reaction volume. (Line 174-177) |
| Priming oligonucleotide (if using GSP) and concentration | E | Random hexamers were used at a concentration of 60 µM. (Line 174-177) |
| Reverse transcriptase and concentration | E | Transcriptor Reverse Transcriptase enzyme was used at the manufacturer-recommended concentration. (Line 174-177) |
| Temperature and time | E | The reverse transcription reaction was carried out at 55°C for 30 minutes, followed by 85°C for 5 minutes. (Line 174-177) |
| Manufacturer of reagents and catalogue numbers | D | Reagents were obtained from Roche, catalog number 04 897 030 001. |
| Cqs with and without RT | D |  |
| Storage conditions of cDNA | D | cDNA was stored at -20°C until further use. |
| **qPCR TARGET INFORMATION** |  |  |
| If multiplex, efficiency and LOD of each assay | E | Multiplex assays were not performed in this study. |
| Sequence accession number | E | Sequence accession numbers for target genes are provided in the supplementary materials. ( Supplementary Table 3). |
| Location of amplicon | D | Amplicons were located within across different exons to avoid amplification of genomic DNA. |
| Amplicon length | E | Amplicon lengths ranged from 86 to 156 bp, and the amplicon sizes of the primers are provided in the supplementary materials (Supplementary Table 3). |
| In silico specificity screen (BLAST, etc) | E | Primers were designed using Primer-BLAST to ensure specificity. |
| Pseudogenes, retropseudogenes or other homologs? | D | Pseudogenes and homologs were checked through BLAST analysis. |
| Sequence alignment | D | Primer sequences were aligned with the reference genome to confirm their specificity. |
| Secondary structure analysis of amplicon | D | Secondary structures were assessed using mFold. |
| Location of each primer by exon or intron (if applicable) | E | Genomic DNA contamination was prevented by using the gDNA Eliminator column during RNA isolation. Primers were designed to span exon-exon junctions, with the intronic regions between exons selected to be large enough to avoid amplification of genomic DNA. |
| What splice variants are targeted? | E | In this study, the largest transcript or the most representative transcript variant for each gene was targeted, which includes the alternative splicing variants. The analysis focused on mRNA expression levels of these transcripts, without distinguishing between individual splice variants. |
| **qPCR OLIGONUCLEOTIDES** |  |  |
| Primer sequences | E | Primer sequences were provided in the supplementary materials (Supplementary Table 3). |
| RTPrimerDB Identification Number | D | Not applicable for this study. |
| Probe sequences | D | Probe sequences were not required as SYBR Green chemistry was utilized. |
| Location and identity of any modifications | E | No modifications were used. |
| Manufacturer of oligonucleotides | D | Primers were synthesized by [Oligomer biotechnolgy], a national company |
| Purification method | D | Primers were purified using HPLC purification. |
| **qPCR PROTOCOL** |  |  |
| Complete reaction conditions | E | Reactions were conducted in a final volume of 20 µL, containing 10 µL of SYBR Green Master Mix, 1 µL of each primer (10 µM), 2 µL of cDNA, and 6 µL of nuclease-free water. |
| Reaction volume and amount of cDNA/DNA | E | 2 µL of cDNA was used in a 20 µL reaction volume. (Line 177-182) |
| Primer, (probe), Mg++ and dNTP concentrations | E | SYBR Green I Master Mix (Roche) was used for qRT-PCR, and the reaction was performed following the manufacturer's protocol, as the mix contains optimized concentrations of Mg++, dNTPs, and enzymes. |
| Polymerase identity and concentration | E | SYBR Green I Master Mix (Roche) was used for qRT-PCR, and the reaction was performed following the manufacturer's protocol. The ready-to-use hot start PCR mix contains FastStart Taq DNA Polymerase, reaction buffer, dNTP mix (with dUTP instead of dTTP), SYBR Green I dye, and MgCl₂, all at optimized concentrations. |
| Buffer/kit identity and manufacturer | E | SYBR® Green I Master Mix from Roche was used. (Line 177-182) |
| Exact chemical constitution of the buffer | D | The ready-to-use hot start PCR mix contains FastStart Taq DNA Polymerase, reaction buffer, dNTP mix (with dUTP instead of dTTP), SYBR Green I dye, and MgCl₂, all at optimized concentrations. |
| Additives (SYBR Green I, DMSO, etc) | E | SYBR Green I was included in the Master Mix. |
| Manufacturer of plates/tubes and catalog number | D | LightCycler® 480 Multiwell 96-Well Plates and sealers were obtained from Roche. |
| Complete thermocycling parameters | E | The PCR cycling conditions included an initial denaturation at 95°C for 3 minutes, followed by 40 cycles of 95°C for 90 seconds, 60°C for 30 seconds, and 72°C for 30 seconds.(Line 179-180). |
| Reaction setup (manual/robotic) | D | Reaction setup was performed manually using pipettes. |
| Manufacturer of qPCR instrument | E | qPCR was conducted using the LightCycler480 instrument (Roche, Basel, Switzerland) in accordance with the manufacturer’s protocol. (Line 178-179). |
| **qPCR VALIDATION** |  |  |
| Evidence of optimisation (from gradients) | D | Gradient PCR was performed to determine the optimal annealing temperature for primer optimization, ensuring efficient amplification and minimal non-specific binding. |
| Specificity (gel, sequence, melt, or digest) | E | Specificity of the primers was confirmed by melting curve analysis using LightCycler 480 software, and, when necessary, validated by gel electrophoresis of PCR products to confirm the expected amplicon size. |
| For SYBR Green I, Cq of the NTC | E | For SYBR Green I, the Cp value of the non-template control (NTC) was greater than 35, confirming the absence of contamination. |
| Standard curves with slope and y-intercept | E | Standard curves were not generated, as the analysis was conducted using the ΔΔCt method, which does not require the construction of a standard curve. |
| PCR efficiency calculated from slope | E | PCR efficiency was not calculated based on a standard curve, as the analysis utilized the ΔΔCt method, which does not require standard curve construction. |
| Confidence interval for PCR efficiency or standard error | D | The PCR efficiency was not directly calculated using a standard curve. Therefore, the confidence interval or standard error of the PCR efficiency was not determined. |
| r2 of standard curve | E | Since a standard curve was not used for this analysis, the r² value is not applicable. |
| Linear dynamic range | E | The linear dynamic range was not assessed using a standard curve, as the ΔΔCt method was applied in this study." |
| Cq variation at lower limit | E | Cp variation at the lower limit was not a concern in this study, as only upregulated genes with relatively high expression levels were analyzed, and no issues were observed with Cq values at low expression. |
| Confidence intervals throughout range | D | Since a standard curve was not generated and the analysis was performed using the delta-delta Ct method, confidence intervals for the entire range were not explicitly calculated. However, the assays were optimized for high specificity and reproducibility, ensuring reliable results within the measured expression levels. |
| Evidence for limit of detection | E | The limit of detection was not explicitly determined in this study since the analysis was focused on highly expressed upregulated genes. No issues related to sensitivity or detection were observed, and all measured genes showed detectable expression levels within the dynamic range of the assay. |
| If multiplex, efficiency and LOD of each assay | E | Multiplex assays were not performed in this study. |
| **DATA ANALYSIS** |  |  |
| qPCR analysis program (source, version) | E | qPCR analysis was performed using Roche LightCycler 480 software 1.5.0 (Line 177-182). |
| Cq method determination | E | Cp values were determined using the crossing point method, as provided by the LightCycler 480 software. (Line 177-182). |
| Outlier identification and disposition | E | Outlier removal was not performed. Triplicate measurements were taken for each sample, and assays were repeated if the results showed significant variability. (Line 177-182). |
| Results of NTCs | E | NTC (No Template Control) results confirmed no contamination throughout the experiments. Strict precautions were taken to prevent contamination, including careful handling of primers and reagents, as well as performing the experiments in a Class II biosafety cabinet. |
| Justification of number and choice of reference genes | E | Reference genes were chosen for their consistent expression across all samples, and at least two reference genes were used in each analysis to ensure reliable normalization. (Line 177-182). |
| Description of normalization method | E | Normalization was carried out using the geometric mean of HPRT and B2M reference genes to account for variations in RNA quantity and quality. (Line 177-182). |
| Number and concordance of biological replicates | D | The study included two separate cohorts: a discovery cohort with 49 and a validation cohort with 64 matched tumor and normal FFPE samples. Matched samples were used to ensure accurate comparisons between tumor and normal tissues from the same individuals, minimizing individual genetic variability. （Line77-84） |
| Number and stage (RT or qPCR) of technical replicates | E | Each sample was tested in triplicate technical replicates during both the reverse transcription (RT) and qPCR stages. |
| Repeatability (intra-assay variation) | E | Intra-assay variation (repeatability) was less than 2%. |
| Reproducibility (inter-assay variation, %CV) | D | Inter-assay variation was determined to be less than 5%. |
| Power analysis | D | The required sample size was determined using power analysis with a statistical power of 0.8. |
| Statistical methods for result significance | E | Statistical significance was determined using a t-test to compare the two groups. A p-value of less than 0.05 was considered statistically significant. (Lines 218-224) |
| Software (source, version) | E | Data analysis was conducted using GraphPad Prism software, version 6.6. (Lines 218-224) |
| Cq or raw data submission using RDML | D |  |
